# NF-κB is a critical mediator of post-mitotic senescence in oligodendrocytes and subsequent white matter loss

**DOI:** 10.1186/s13024-023-00616-5

**Published:** 2023-04-17

**Authors:** Judith Stefanie Schlett, Melanie Mettang, Aladdin Skaf, Pavel Schweizer, Alina Errerd, Ephraim Alemayehu Mulugeta, Tabea Melissa Hein, Konstantinos Tsesmelis, Miltiadis Tsesmelis, Ulrike F. G. Büttner, Heinrich Wendt, Alireza Abaei, Volker Rasche, Vivien Prex, Ester Nespoli, Najwa Ouali Alami, Daniel Tews, Paul Walther, Deniz Yilmazer-Hanke, Franz Oswald, Leda Dimou, Thomas Wirth, Bernd Baumann

**Affiliations:** 1 Institute of Physiological Chemistry, Ulm University, Albert- Einstein-Allee 11, 89081 Ulm, Germany; 2 Molecular and Translational Neuroscience, Department of Neurology, University Medical Center Ulm, 89081 Ulm, Germany; 3 Core Facility Small Animal Imaging (CF-SANI), Ulm University, 89081 Ulm, Germany; 4 Institute of Clinical Neuroanatomy, Ulm University, Helmholtzstraße 8/1, 89081 Ulm, Germany; 5 Core Facility Extracellular Flux Analyzer, Ulm University Medical Center, 89081 Ulm, Germany; 6 Central Facility for Electron Microscopy, Ulm University, 89081 Ulm, Germany; 7 Department of Internal Medicine I, Center for Internal Medicine, University Medical Center Ulm, 89081 Ulm, Germany

**Keywords:** White matter degeneration, Mature oligodendrocytes, Inflammaging, NF-κB, PoMICS, Integrated stress response

## Abstract

**Background:**

Inflammaging represents an accepted concept where the immune system shifts to a low-grade chronic pro-inflammatory state without overt infection upon aging. In the CNS, inflammaging is mainly driven by glia cells and associated with neurodegenerative processes. White matter degeneration (WMD), a well-known process in the aging brain, manifests in myelin loss finally resulting in motor, sensory and cognitive impairments. Oligodendrocytes (OL) are responsible for homeostasis and maintenance of the myelin sheaths, which is a complex and highly energy demanding process sensitizing these cells to metabolic, oxidative and other forms of stress. Yet, the immediate impact of chronic inflammatory stress like inflammaging on OL homeostasis, myelin maintenance and WMD remains open.

**Methods:**

To functionally analyze the role of IKK/NF-κB signaling in the regulation of myelin homeostasis and maintenance in the adult CNS, we established a conditional mouse model allowing NF-κB activation in mature myelinating oligodendrocytes. IKK2-CA^PLP−CreERT2^ mice were characterized by biochemical, immunohistochemical, ultrastructural and behavioral analyses. Transcriptome data from isolated, primary OLs and microglia cells were explored by in silico pathway analysis and validated by complementary molecular approaches.

**Results:**

Chronic NF-κB activation in mature OLs leads to aggravated neuroinflammatory conditions phenocopying brain inflammaging. As a consequence, IKK2-CA^PLP−CreERT2^ mice showed specific neurological deficits and impaired motoric learning. Upon aging, persistent NF-κB signaling promotes WMD in these mice as ultrastructural analysis revealed myelination deficits in the corpus callosum accompanied by impaired myelin protein expression. RNA-Seq analysis of primary oligodendrocytes and microglia cells uncovers gene expression signatures associated with activated stress responses and increased post mitotic cellular senescence (PoMiCS) which was confirmed by elevated senescence-associated β-galactosidase activity and SASP gene expression profile. We identified an elevated integrated stress response (ISR) characterized by phosphorylation of eIF2α as a relevant molecular mechanism which is able to affect translation of myelin proteins.

**Conclusions:**

Our findings demonstrate an essential role of IKK/NF-κB signaling in mature, post-mitotic OLs in regulating stress-induced senescence in these cells. Moreover, our study identifies PoMICS as an important driving force of age-dependent WMD as well as of traumatic brain injury induced myelin defects.

**Graphical Abstract:**

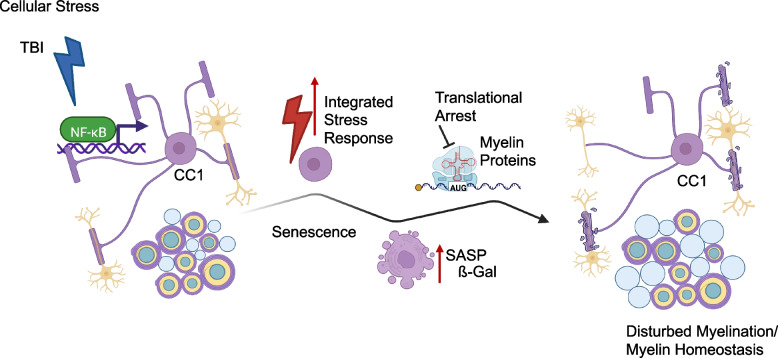

**Supplementary Information:**

The online version contains supplementary material available at 10.1186/s13024-023-00616-5.

## Background

Aging includes a decline in brain function leading to deficits in motor, sensory and cognitive performance. Imaging studies in humans and other mammals document a volume shrinkage of the aging brain with reduction in both grey and white matter substance, and *post mortem* brain analyses identified widespread myelin defects in aged humans [1–6].

The causes include degenerative changes of myelin sheaths with subsequent axonal loss, overall compromising the conduction velocity and leading to dysfunctional neuronal circuits [7]. Myelination and homeostatic maintenance of myelin sheaths critically depend on the function of both mature OLs and their progenitor cells (called OPCs or NG2-glia), a process coupled to high energy demand. OL possess only limited capacity of self-protection and are prone to mitochondrial dysfunction leading to oxidative stress, an issue, often observed in the context of neuroinflammatory diseases [8]. Thus, aging-dependent white matter loss in humans and in mouse models argues for a stress-induced deregulation of myelination and/or deficits in the maintenance of healthy myelin sheaths [4–6, 9].

The IKK/NF-κB signaling pathway is a well-known mediator of inflammatory and stress responses [10–12], also including the regulation of diverse neuroinflammatory reactions [13–15]. Various age-related diseases are all linked to NF-κB mediated inflammatory processes [16, 17] and chronic neuroinflammation is increasingly recognized as a central pathogenic mechanism in neurodegeneration [18]. IKK/NF-κB signaling is acting as a major driver of aging processes in different organs including the brain [19, 20], and is also involved in or even causes cellular senescence [21–23] and regulates the SASP secretome [24, 25]. Moreover, progeroid mouse models based on permanent genotoxic stress share an upregulation of NF-κB signaling [26, 27]. Conversely, genetic or pharmacological inhibition of NF-κB delays the onset of phenotype development or mitigates the age-related disease burden indicating a promising translational role of NF-κB in aging processes [28–30]. However, the underlying cellular and molecular mechanisms as well as the cellular specificity of NF-κB initiated aging processes in the brain still remain elusive. In addition, IKK/NF-κB signaling is a major regulator of inflammaging, a persistent, sterile and gradually progressing inflammatory condition also acting in the CNS [31]. The driving force of inflammaging are different forms of cellular stress like the accumulation of molecules summarized as damage-associated molecular patters (DAMPs), which are all competent to activate the IKK/NF-κB pathway. However, the mechanistic interrelations connecting inflammation, cellular stress, OL function and WMD still remain elusive.

While developmental myelination is independent of NF-κB function, we asked whether IKK/NF-κB signaling is involved in the aging-dependent regulation of myelin homeostasis and maintenance and how NF-κB mediated inflammaging in OLs may contribute to WMD.

## Results

### NF-κB activation in Oligodendrocytes initiates inflammatory conditions in the CNS

**Fig. 1:**
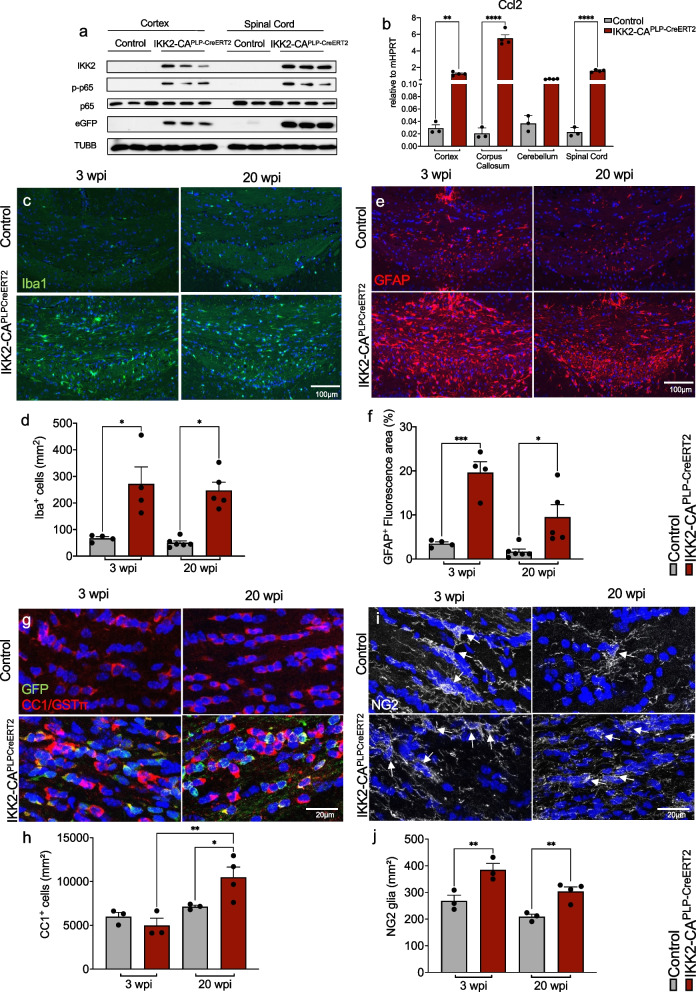
IKK2/NF-κB activation in oligodendrocytes induces an inflammatory phenotype in the CNS. **a** Immunoblot analysis of extracts from cortex and spinal cord tissue from control and IKK2-CA^PLP−CreERT2^ mice 3 weeks post induction (wpi). Strong expression of IKK2 and eGFP demonstrate tamoxifen-dependent transgene activation in IKK2-CA^PLP−CreERT2^ mice. High levels of phosphorylated p65 (p-p65) validate functional activation of NF-κB signaling upon transgene expression (*n* = 3). **b** Gene expression analysis of Ccl2 as an inflammatory marker and NF-κB target gene in tissue extracts from cortex, corpus callosum, cerebellum and spinal cord proves functionality of the transgenic system and depicts the inflammatory milieu within the CNS (*n* = 3–4). **c** and **d** Immunofluorescent staining and quantification of Iba1 reveals persistent microgliosis in the CC (3 wpi – Control (*n* = 4): 67 ± 6 cells/mm^2^, IKK2-CA^PLP−CreERT2^ (*n* = 4): 271 ± 64 cells/mm^2^, 20 wpi—Control (*n* = 6): 50 ± 7 cells/mm^2^, IKK2-CA^PLP−CreERT2^ (*n* = 5): 247 ± 31 cells/mm^2^). **e** and **f** Immunofluorescent staining and quantification of GFAP + astrocytes identifies an increased number of GFAP + astrocytes in the CC at 3 wpi that declines at 20 wpi (3 wpi – Control (*n* = 4): 3.50% ± 0.34 fluorescent area, IKK2-CA^PLP−CreERT2^ (*n* = 4): 19.62% ± 2.13 fluorescent area; 20wpi – Control (*n* = 6): 1.67% ± 0.53 fluorescent area, IKK2-CA^PLP−CreERT2^ (*n* = 5): 9.50% ± 2.53 fluorescent area). **g** and **h** Immunohistochemical analysis of oligodendrocytes (OLs) 3 and 20 wpi shows a significant increase in the overall CC1 + cell number in the corpus callosum (CC) of IKK2-CA^PLP−CreERT2^ animals at 20 wpi with a constant number of GFP + CC1 + cells (3 wpi—Control (*n* = 3): 5997 ± 398 cells/mm^2^, IKK2-CA^PLP−CreERT2^ (*n* = 3): 4979 ± 685 cells/mm^2^; 20 wpi – Control (*n* = 3): 7123 ± 147 cells/mm^2^, IKK2-CA^PLP−CreERT2^ (*n* = 4): 10,484 ± 1014 cells/mm^2^; GFP + cells- IKK2-CA^PLP−CreERT2^: 3 wpi: 3141 ± 575 cells/mm^2^, 20 wpi: 3883 ± 434 cells/mm^2^). **i** and **j** Immunohistochemical analysis of NG2 glia at 3 and 20 wpi reveals higher numbers of progenitor cells in IKK2-CA^PLP−CreERT2^ mice. White Arrows indicate NG2 glia. (3 wpi—Control (*n* = 3): 268 ± 17 cells/mm^2^, IKK2-CA^PLP−CreERT2^ (*n* = 3): 385 ± 20 cells/mm^2^; 20 wpi – Control (*n* = 3): 209 ± 8 cells/mm^2^, IKK2-CA^PLP−CreERT2^ (*n* = 4): 304 ± 15 cells/mm^2^). Data are shown as mean ± SEM. Grey bars, control; red bars, IKK2-CA^PLP−CreERT2^. Statistical analysis: One-way or Two-way ANOVA multiple comparison test followed by Bonferroni´s post hoc test (*p < 0.05; **p < 0.01; ***p < 0.001; ****p < 0.0001 NS, non-significant (*p* > 0.05))

To elucidate the role of NF-κB in the regulation of myelin homeostasis and maintenance we established a conditional OL-specific IKK/NF-κB gain-of-function model named IKK2-CA^PLP−CreERT2^ allowing tamoxifen-dependent transgene expression in around 60% of CC1-positive OLs in adult mice (Fig. S1a-d). Immunoblot analysis revealed significant upregulation of IKK2-CA transgene and eGFP reporter gene expression in different CNS areas of adult IKK2-CA^PLP−CreERT2^ mice 3 weeks after tamoxifen treatment (3 wpi), and p65 phosphorylation confirmed functional activation of the canonical IKK/NF-κB signaling pathway (Fig. 1a and Fig. S1e). As consequence various neuroinflammation marker genes, like *Ccl2* or *Ccl5* were significantly upregulated in different areas (Fig. 1b and Fig. S1f), while other genes like *iNos, IL1β*, or *Lcn2* show a rather selective upregulation in the OL-rich corpus callosum (Fig. S1g). Notably, we detected reactive gliosis in response to NF-κB-mediated gene expression in OLs as prominent Iba1 and GFAP staining identified persisting microgliosis and rather transient astrogliosis in the corpus callosum when analyzed at 3 and 20 wpi (Fig. 1c-f).

In the healthy adult brain, homeostasis of NG2-glia/OPCs and OLs is strictly controlled [32] but gets disturbed in models of brain injury and neurodegenerative disease [33]. Interestingly, the number of mature CC1 + OLs in the corpus callosum significantly increased at 20 wpi in IKK2-CA^PLP−CreERT2^ mice but was found similar at 3 wpi whereas the absolute number of OLs with GFP reporter gene expression did not change between 3 and 20 wpi (Fig. 1g and h). Conversely, an overall increased number of NG2-glia/OPCs was determined at both time points (Fig. 1i and j), but mitotic NG2-glia/OPCs (Pdgfrα/Ki67) were only present at 3 wpi (Fig. S1h and i). Importantly, the lack of NG2/GFP double positive cells (Fig. S1j) argues against transgene activity in NG2-glia/OPCs thereby excluding direct NF-κB-mediated effects on NG2 cells. Similar, the transgene is not active in the peripheral nervous system as GFP expression was absent in sciatic nerve tissue (Fig. S1k). Thus, NF-κB activation in mature, myelinating CC1 + OLs is sufficient to induce reactive gliosis and non-cell-autonomous expansion of NG2-glia/OPCs leading to an increase in CC1 + OLs number. This is further supported by the absence of apoptotic OLs determined by TUNEL-assay at 3 and 20 wpi (data not shown).

### IKK2-CA^PLP−CreERT2^animals develop neurological deficits and decreased motoric learning

**Fig. 2:**
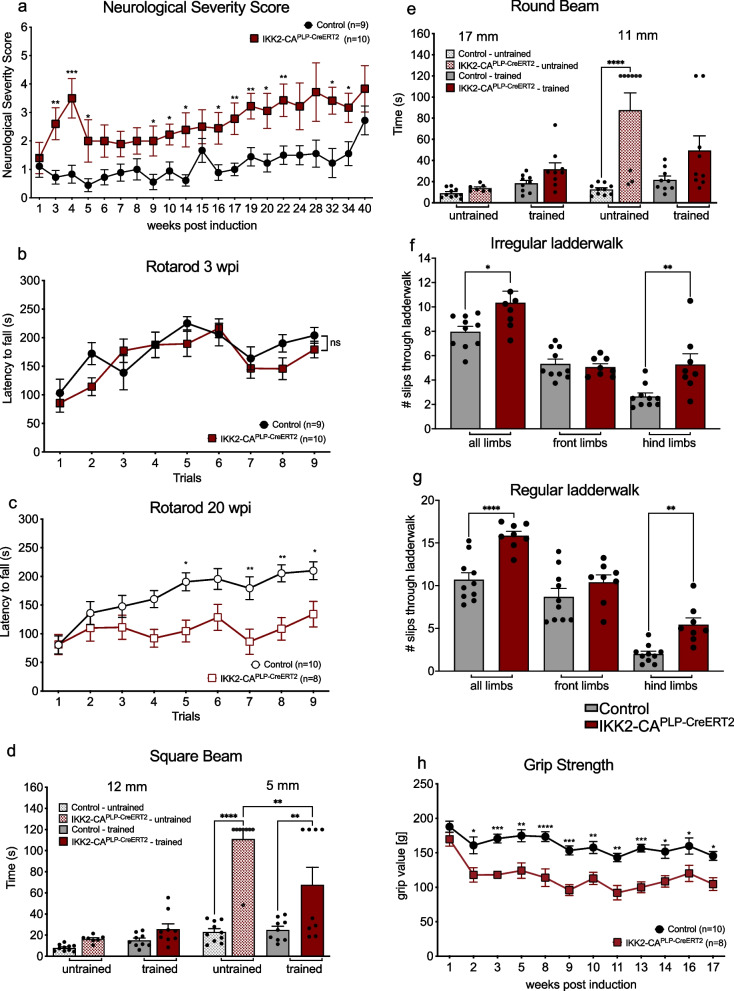
IKK2-CA^PLP−CreERT2^ mice develop neurological deficits and show decreased motoric learning. **a** IKK2-CA^PLP−CreERT2^ mice exhibit an increased neurological severity score (NSS) over the time course of 40 weeks, peaking at 3 to 4 wpi. **b** 9 training trials on the rotarod on 3 consecutive days (3 trials per day) immediately after tamoxifen withdrawal show no difference in rotarod performance between IKK2-CA^PLP−CreERT2^ and control littermates. **c** Constant NF-κB activation in OLs significantly affects rotarod performance. At 20 wpi, IKK2-CA^PLP−CreERT2^ animals show a significantly decreased latency to fall off the rotarod, when first trained at this timepoint. Furthermore, animals do not improve with trial number within the 9 trials on 3 consecutive days (3 trials per day) compared to controls. **d** and **e** Constant training attenuates motor deficits in IKK2-CA^PLP−CreERT2^ mice. Mice were either introduced right after transgene induction and then trained for 20 weeks bi-weekly in the beam walking test (“trained”) or introduced to the beam walking test at 20 wpi (“untrained”). No differences were found in easier tasks like crossing the 12 mm square and 17 mm round beam. Untrained and trained control and IKK2-CA^PLP−CreERT2^ mice mastered the beam walking task in comparable times (untrained: 12 mm: Control: 8 ± 0.9 s, IKK2-CA^PLP−CreERT2^: 16 ± 1.2 s, 17 mm: Control: 9 ± 1.3 s, IKK2-CA^PLP−CreERT2^: 14 ± 1.1 s, trained: 12 mm: Control: 15 ± 2.0 s, IKK2-CA^PLP−CreERT2^: 26 ± 5.3 s, 17 mm: Control: 18 ± 2.6 s, IKK2-CA^PLP−CreERT2^: 32 ± 6.5 s, *n* = 7–10). For smaller and therefore more difficult beams, 5 mm square or 11 mm round beam, untrained IKK2-CA^PLP−CreERT2^ animals take significantly longer to cross the beam than untrained control mice. Crossing times of trained and untrained controls are similar. Trained IKK2-CA^PLP−CreERT2^ animals cross the 5 mm beam in significantly less time compared to untrained IKK2-CA^PLP−CreERT2^ animals, no difference was found between trained control and IKK2-CA^PLP−CreERT2^ animals on the 11 mm beam (untrained: 5 mm: Control: 23 ± 2.9 s, IKK2-CA^PLP−CreERT2^: 111 ± 8.9 s, 11 mm: Control: 12.5 ± 1.5 s, IKK2-CA^PLP−CreERT2^: 87.5 ± 17.2 s, trained: 5 mm: Control: 25 ± 3.3 s, IKK2-CA^PLP−CreERT2^: 61.1 ± 17.3 s, 11 mm: Control: 21.6 ± 3.4 s, IKK2-CA^PLP−CreERT2^: 49.3 ± 14.8 s, *n* = 7–10). **f** and **g** The ladderwalk test revealed distinct motoric deficits 20 wpi. IKK2-CA^PLP−CreERT2^ animals showed significantly more missteps in the irregular as well as in the regular ladderwalk there to even greater extent. Missteps are mainly shown in the hindlimbs (irregular: all limbs: Control: 8.0 ± 0.38 missteps, IKK2-CA^PLP−CreERT2^: 10.3 ± 0.84 missteps, front limbs: Control: 5.3 ± 0.35 missteps, IKK2-CA^PLP−CreERT2^: 5.1 ± 0.23 missteps, hind limbs: Control: 2.7 ± 0.27 missteps, IKK2-CA^PLP−CreERT2^: 5.3 ± 0.77 missteps, regular: all limbs: Control: 10.7 ± 0.74 missteps, IKK2-CA^PLP−CreERT2^: 15.8 ± 0.46 missteps, front limbs: Control: 8.7 ± 0.87 missteps, IKK2-CA^PLP−CreERT2^: 10.4 ± 0.75 missteps, hind limbs: Control: 2.0 ± 0.30 missteps, IKK2-CA^PLP−CreERT2^: 5.4 ± 0.70 missteps, *n* = 8–10). **h** Grip strength was measured up to 17 wpi revealing significantly decreased grip strength in IKK2-CA^PLP−CreERT2^ mice. Data are shown as mean ± SEM. Black dots/grey bars, control; red dots/bars, IKK2-CA^PLP−CreERT2^; striped bars: untrained controls and IKK2-CA^PLP−CreERT2^. Statistical analysis: Two-way ANOVA multiple comparison test followed by Bonferroni´s post hoc test (*p < 0.05; **p < 0.01; ***p < 0.001; ****p < 0.0001 NS, non-significant (*p* > 0.05))

IKK2-CA^PLP−CreERT2^ mice show a normal weight development until 19 wpi, however thereafter weight gain is significantly decreased compared to controls (Fig. S2a). To address the functional consequences of chronic IKK/NF-κB signaling in OLs, we conducted long-term behavioral analyses including Neurological Severity Score [34] (NSS), rotarod, beam walk, ladder walk as well as grip strength measurement. Significant neurological deficits were discovered already 3 wpi, and over the whole course of 40 weeks IKK2-CA^PLP−CreERT2^ animals showed higher scores in the NSS than control animals (Fig. 2a). When animals were introduced to the rotarod task immediately after transgene activation, there was no difference in the motoric performance of IKK2-CA^PLP−CreERT2^ and control animals (Fig. 2b) until 40 wpi (Fig. S2b). However, IKK2-CA^PLP−CreERT2^ animals trained for the first time at the 20 wpi timepoint failed to improve their performance over the course of the trials (Fig. 2c). A similar picture appeared in the beam walking test with one group trained continuously for 20 weeks (“trained”) and another group that was introduced to the task for the first time at 20 wpi (“untrained”). Both groups managed the easier tasks (12 mm square and a 17 mm round beam) similarly, however for the more difficult tasks (5 mm square and 11 mm round beam) untrained transgenic animals needed significantly longer to cross the beam. This deficit was significantly attenuated, when animals had been continuously trained for 20 weeks (Fig. 2d and e) implying that aged IKK2-CA^PLP−CreERT2^ animals have a decreased capacity to learn new motor skills. Next, the ladder walk test was employed to assess motor coordination [35]. IKK2-CA^PLP−CreERT2^ animals exhibited significantly more missteps both in the irregular as well as in the regular ladderwalk with even greater difference in the latter (Fig. 2f and g). Importantly, we could not determine any obvious cerebellar atrophy or degeneration of purkinje cells (Fig. S2c-e), as previously observed in mouse model with chronic NF-κB activation in astrocytes [36]. Finally, grip strength measurement revealed a significant lower performance already early after transgene activation which does not further increase with aging (Fig. 2h).

These data suggest, that IKK2-CA^PLP−CreERT2^ mice develop a specific motoric phenotype resembling a disruption of white matter integrity linked with poor motor performance.

### IKK/NF-κB activation in oligodendrocytes promotes white matter loss

**Fig. 3:**
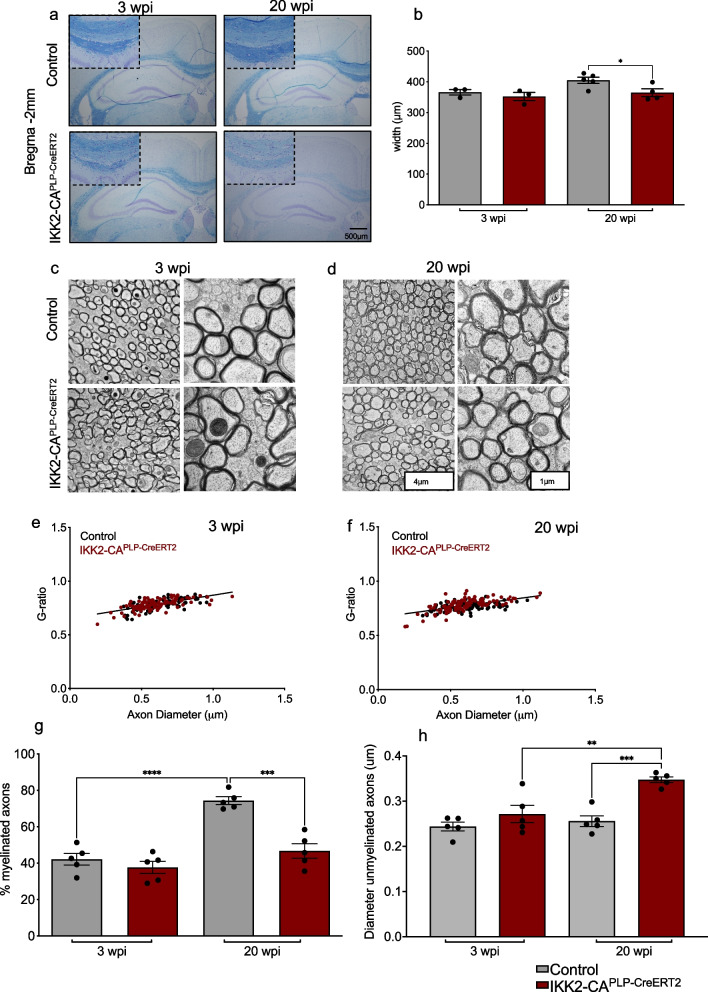
Chronic NF-κB activation in OLs leads to white matter loss in the corpus callosum. **a** and **b** Luxol Fast Blue (LFB) staining revealed a significant decrease (B) in the width of the CC in IKK2-CA^PLP−CreERT2^ animals at 20 wpi (Bregma—2 mm; 3wpi: Control: 366 ± 9.0 μm, IKK2-CA^PLP−CreERT2^: 352 ± 13.3 μm; 20wpi: Control: 405 ± 10.0 μm, IKK2-CA^PLP−CreERT2^: 364.6 ± 12.6 μm, *n* = 3–4). **c** to **f** Myelinated axons do not show differences in myelin sheath structure. Ultrastructural analysis using electron microscopy reveals proper myelination of single axons in the CC (**c** and **d**). G-ratio, the ratio of the inner and outer diameter of the myelin sheath used as reference for optimal myelination, did not differ between IKK2-CA^PLP−CreERT2^ and control animals at both time points (**e** and **f**). **g** NF-κB activation in OLs affects overall axonal myelination. The percentage of myelinated axons is significantly decreased in the CC of IKK2-CA^PLP−CreERT2^ animals at 20 wpi. Number of myelinated axons remain at the level of 3 wpi in IKK2-CA^PLP−CreERT2^ mice (3 wpi: Control: 42.1 ± 3.2%, IKK2-CA^PLP−CreERT2^: 37.7 ± 3.6%; 20 wpi: Control: 74.4 ± 2.1%, IKK2-CA^PLP−CreERT2^: 46.7 ± 4.0%, *n* = 5). **h** Axon diameter of unmyelinated axons is significantly increased in the CC of IKK2-CA^PLP−CreERT2^ animals 20 wpi in comparison to IKK2-CA^PLP−CreERT2^ animals at 3 wpi as well as controls (3 wpi: Control: 0.24 ± 0.01 μm, IKK2-CA^PLP−CreERT2^: 0.27 ± 0.02 μm; 20 wpi: Control: 0.25 ± 0.01 μm, IKK2-CA^PLP−CreERT2^: 0.35 ± 0.01 μm, *n* = 5). Data are shown as mean ± SEM. Black dots/grey bars, control; red dots/bars, IKK2-CA.^PLP−CreERT2^. Statistical analysis: Two-tailed Mann–Whitney-U Test, Two-way ANOVA multiple comparison test followed by Bonferroni´s post hoc test, (*p < 0.05; **p < 0.01; ***p < 0.001; ****p < 0.0001 NS, non-significant (*p* > 0.05))

Intact myelin sheath formation and maintenance of its integrity is an important prerequisite for learning new and complex motor skills [37]. Therefore, we examined the myelination status in IKK2-CA^PLP−CreERT2^ mice. Interestingly, we identified a modest but significant degeneration of the corpus callosum at different bregma points using Luxol Fast Blue (LFB) staining at 20 wpi whereas no obvious difference was determined at 3wpi (Fig. 3a and b, Fig. S3g-j). *Ex-vivo* MRI analysis of brains at 20 wpi revealed decompacted white matter tracts, hypointensities and thinning of the corpus callosum (Fig. S3a-c). Subsequently, electron microscopy was exploited for ultrastructural analysis of myelin sheaths revealing that g-ratio, appearance and variance in the number and diameter of myelinated axons was not altered at 3 wpi and 20 wpi. (Fig. 3c-f, Fig. S3d and e). However, the overall percentage of myelinated axons was significantly reduced in IKK2-CA^PLP−CreERT2^ animals at 20 wpi (Fig. 3g). At this timepoint, the degree of axonal myelination just remained at the level of 3 wpi whereas the known aging-dependent increase in myelination was detected in controls. Usually, CNS axons become myelinated at a diameter size larger than around 0.2 μm [38]. This principle gets disturbed in the IKK2-CA^PLP−CreERT2^ model as we found a significant increase in the size of unmyelinated axons at 20 wpi (Fig. 3h und S3f). However, we did not observe obvious early axonal pathology by SMI32 staining (Fig. S2f).

### Myelination and myelin homeostasis associated gene expression is only marginally affected by NF-κB activation in oligodendrocytes

**Fig. 4:**
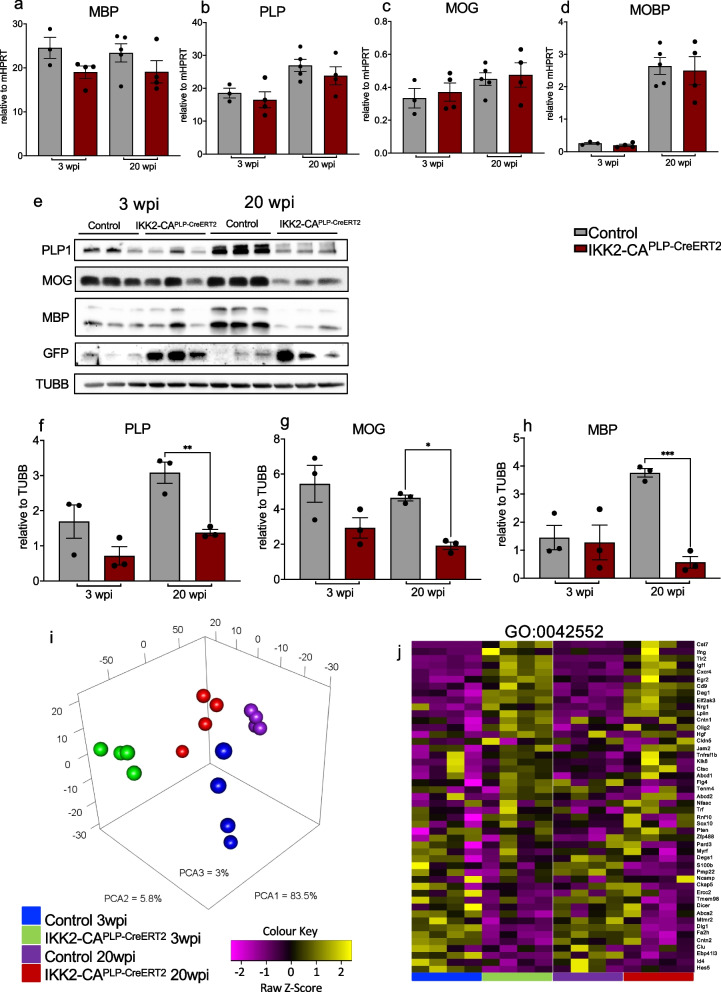
NF-κB activation in OLs impairs myelin-associated protein expression. **a** to **d** qRT-PCR reveals no differences in gene expression of *Mbp* (**a**), *Plp* (**b**), *Mog* (**c**) and *Mobp* (**d**) at 3 and 20 wpi in cortex tissue (*n* = 3–5). **e** to **h** Western blot analysis of cortex tissue indicates significantly decreased protein levels of MBP, MOG and PLP1 at 20 wpi in IKK2-CA^PLP−CreERT2^ mice, while protein levels at 3 wpi were comparable between control and IKK2-CA^PLP−CreERT2^ animals (*n* = 3). **i** Principal component analysis of isolated O4^+^ OLs (RNA sequencing) at 3 and 20 wpi shows distinct clustering within control and IKK2-CA^PLP−CreERT2^ groups (*n* = 4, two brains with identical genotype were pooled to *n* = 1). **j** Transcriptome analysis determined mild gene expression changes between IKK2-CA^PLP−CreERT2^ and control O4^+^ OLs in genes related to to the GO class (GO:0,042,552) myelination, myelin assembly and maintenance, regulation of myelination and paranodal junction assembly. Out of all genes depicted in the heatmap only *Cst7, Tlr2, Igf1, Eif2ak3, Cxcr4, Lpin1, Egr2, Cd9, Dag1*, and *Epb41l3* were significantly deregulated at both timepoints. At 3wpi additionally *Ifng, Hes 5, Cntn2* and *Dlg1* reached significance, at 20 wpi *Nrg1, Id4, Tmem98* and *S100b* were found significantly deregulated in addition. Data are shown as mean ± SEM. Grey bars, control; red bars, IKK2-CA^PLP−CreERT2^. Statistical analysis: Two-way ANOVA multiple comparison test followed by Bonferroni´s post hoc test, (*p < 0.05; **p < 0.01; ***p < 0.001; ****p < 0.0001 NS, non-significant (*p* > 0.05))

To gain further insight into this specific myelination defect, we next conducted gene and protein expression analysis of myelination-relevant genes. Whole cortex tissue revealed no significant changes in *Mbp*, *Plp, Mog* and *Mobp* mRNA expression at 3 wpi and 20 wpi. In contrast, Mbp, Plp1 and Mog protein levels were clearly decreased especially at 20 wpi in the cortex (Fig. 4a-h). To understand the mechanism(s) underlying this decline in myelin proteins in detail, we isolated O4^+^ OLs from IKK2-CA^PLP−CreERT2^ animals and control littermates at 3 and 20 wpi. The number of isolated O4^+^ OLs was similar in both groups, and subsequent RNA and protein analyses revealed strong expression of the IKK2-CA transgene and the GFP reporter gene in IKK2-CA^PLP−CreERT2^ O4^+^ cells (Fig. S4a-e). FACS analysis showed that the overall amount of O4^+^ cells within the MACS purified cell population was around 70%. There was a prominent enrichment of GFP^+^ O4 cells, but not all O4 purified cells express GFP similar to the immunohistological analyses shown before (Fig. 1) and according to the restricted PLP-promotor activity of the driver line [39]. Only OLs with an active PLP-promotor (around 60% of CC1 cells) can undergo recombination within the 3-week time frame of tamoxifen application. All OLs differentiating from NG2-glia/OPCs later in the absence of tamoxifen cannot recombine anymore thereby reducing the number of GFP^+^ cells within the isolated O4^+^ fraction. FACS staining of O4-bead sorted cells revealed that around 75% of O4 ^+^ cells are also GalC positive and all cells with high O4 expression are positive for the mature OL marker MOG (Fig. S4g).

RNA sequencing analysis of purified O4^+^ OLs revealed 412/181 significantly down and 1121/602 significantly upregulated genes at 3 and 20 wpi, respectively (Fig. S4h and i). Distinct differences between cells of IKK2-CA^PLP−CreERT2^ and control mice show a clear separation of the groups in the principal component analysis (Fig. 4j). Consistent with the decreased number of GFP^+^ O4^+^ OLs, NF-κB target gene expression at 20 wpi stays significantly upregulated, however overall level decreased compared to 3 wpi (Fig. S4k). Similar to whole cortex tissue analysis (Fig. 4a-d), genes encoding structural proteins involved in myelin sheath formation (GO: 0,043,209) like *Mog, Mbp, Cnp, Mobp* and *Plp1* were slightly affected in O4^+^ OLs from IKK2-CA^PLP−CreERT2^ mice without reaching overall significance (Fig. S4j). Other genes connected to myelination, myelin assembly and maintenance, regulation of myelination and paranodal junction assembly (GO:0,042,552) including *Cst7, Tlr2, Igf1, Epb41l3, Hes5* and *Cntn2* also revealed a mild deregulation pattern without reaching overall significance (Fig. 4j).

These findings imply that IKK/NF-κB signaling in OLs does not directly interfere with transcriptional regulation of genes relevant for myelination but rather affects different cellular processes which as a consequence result in myelin protein loss.

### IKK/NF-κB activation in mature oligodendrocytes induces a specific stress and senescence program eliciting translational arrest

**Fig. 5:**
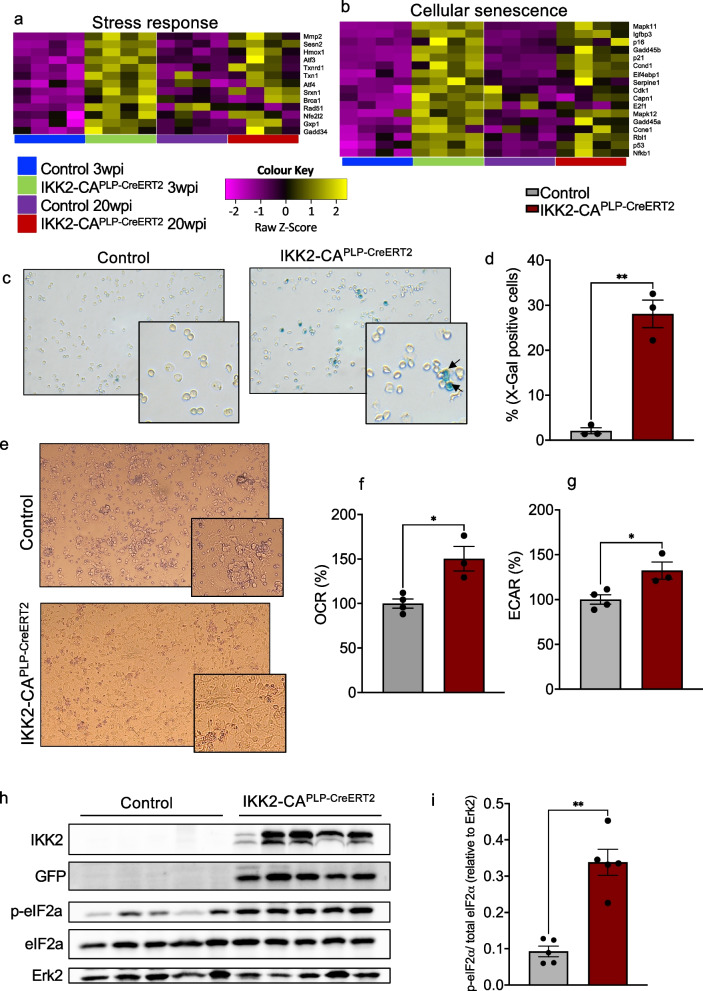
Genes associated with stress responses and cellular senescence are upregulated upon NF-κB activation in OLs. **a** and **b** Heatmaps indicate significantly elevated gene signatures indicative for stress responses (**a**) and cellular senescence (**b**) in IKK2-CA^PLP−CreERT2^ O4^+^ OLs compared to control OLs at 3 and 20 wpi. **c** and **d** X-gal staining revealed significantly increased senescence associated ß-galactosidase activity in freshly isolated IKK2-CA^PLP−CreERT2^ O4^+^ OLs at 3 wpi. Blue X-gal positive cells are indicated by black arrows (Control: 2.1% ± 0.67% X-gal positive cells, IKK2-CA^PLP−CreERT2^: 28.1% ± 3.05% x-gal positive cells, *n* = 3). **e** Primary O4^+^ OLs isolated from IKK2-CA^PLP−CreERT2^ mice show senescence associated cell morphology as they adopt a larger and flattened morphology. **f** and **g** IKK2-CA^PLP−CreERT2^ O4^+^ OLs show an overall increased metabolic activity as oxidative phosphorylation (F, OCR normalized to control; IKK2-CA^PLP−CreERT2^: 150.2 ± 11.9%, *n* = 3–4) as well as glycolysis (G, ECAR normalized to control; IKK2-CA^PLP−CreERT2^: 132.3 ± 8.3%, *n* = 3–4) is significantly increased. **h** NF-κB activation in O4^+^ OLs leads to an increase in phosphorylated eIF2α indicating activation of the integrated stress response as revealed by western blot analysis at 3wpi. Prominent IKK2 and GFP reporter gene expression confirm transgene activation in IKK2-CA^PLP−CreERT2^ cells. **i** Quantification of h (*n* = 5). Data are shown as mean ± SEM. Grey bars, control; red bars, IKK2-CA.^PLP−CreERT2^. Statistical analysis: Two-tailed Mann–Whitney-U Test, (*p < 0.05; **p < 0.01; ***p < 0.001; ****p < 0.0001 NS, non-significant (*p* > 0.05))

To follow the above hypothesis, we applied GSEA to our RNAseq data and detected especially at 3 wpi a prominent enrichment in pathways related to cellular stress, senescence, SASP factors and p53 signaling in IKK2-CA^PLP−CreERT2^ OLs (Fig. S5a-c). This includes profound marker genes involved in oxidative stress like *Hmox 1 or Txn 1*, DNA damage response genes like *Brca1*, marker genes for ER Stress like *ATF4* and *GADD34* and cellular senescence related genes like *GADD45*, *p38 (MAPK11/MAPK12)*, *p16*, *p21* or *p53* (Fig. 5a and b). Recently the term post mitotic cellular senescence (PoMiCS) evolved to include post mitotic, terminally differentiated cells [40]. To test whether the senescence gene expression signature leads to senescent OLs we monitored SA-β-galactosidase activity, a biomarker for cellular senescence. Importantly, we found a strong increase in SA-β-galactosidase positive OLs isolated from IKK2-CA^PLP−CreERT2^ animals compared to controls (Fig. 5c and d). Cellular senescence is typically associated with changes in cell morphology and also characterized by a highly active metabolism with increased glycolytic activity [41]. When primary O4^+^ OLs were cultured and differentiated ex vivo we observed differences in size and morphology compared to O4^+^ OLs isolated from control animals (Fig. 5e). In addition, we found the mitochondrial respiration rate (oxidative consumption rate, OCR, Fig. 5f) as well as glycolysis (extracellular acidification rate, ECAR, Fig. 5g) significantly upregulated in IKK2-CA^PLP−CreERT2^ OLs (3 wpi).

Translational control represents a paradox during senescence of proliferating cells as selective translation of. SASP is promoted while global translation is repressed [42] (Fig. S5c). To characterize the reduction of myelin proteins in IKK2-CA^PLP−CreERT2^ OLs we investigated the regulation of translation in O4^+^ OLs. An important factor involved in this process is the eucaryotic initiation factor 2 (eIF2). Phosphorylation of the α-subunit of eIF2 by stress-activated kinases renders eIF2 function to act as a negative regulator of translation. The overall outcome is a decrease in global protein synthesis and enhancement of translation of stress-related mRNAs (Fig. S5d) [43]. In line with this, prolonged IKK/NF-κB signaling in OLs leads to a significant increase in phosphorylated eIF2α, while absolute levels of eIF2α do not change (Fig. 5h and i). Furthermore, RNA-Seq analysis revealed a significant upregulation of stress response genes like ATF4 by fourfold and the upstream kinase PERK by 1.6-fold. Consistent with increased protein degradation, we detected a significant enrichment of genes involved in proteasome function (Fig. S5e).

Thus, IKK2 activation in post-mitotic OLs is capable of inducing a specific stress response program leading to premature senescence characterized by suppressed myelin protein translation and prominent upregulation of SASP factors.

### IKK/NF-κB activation in oligodendrocytes promotes microglia polarization towards aged white matter microglia

**Fig. 6:**
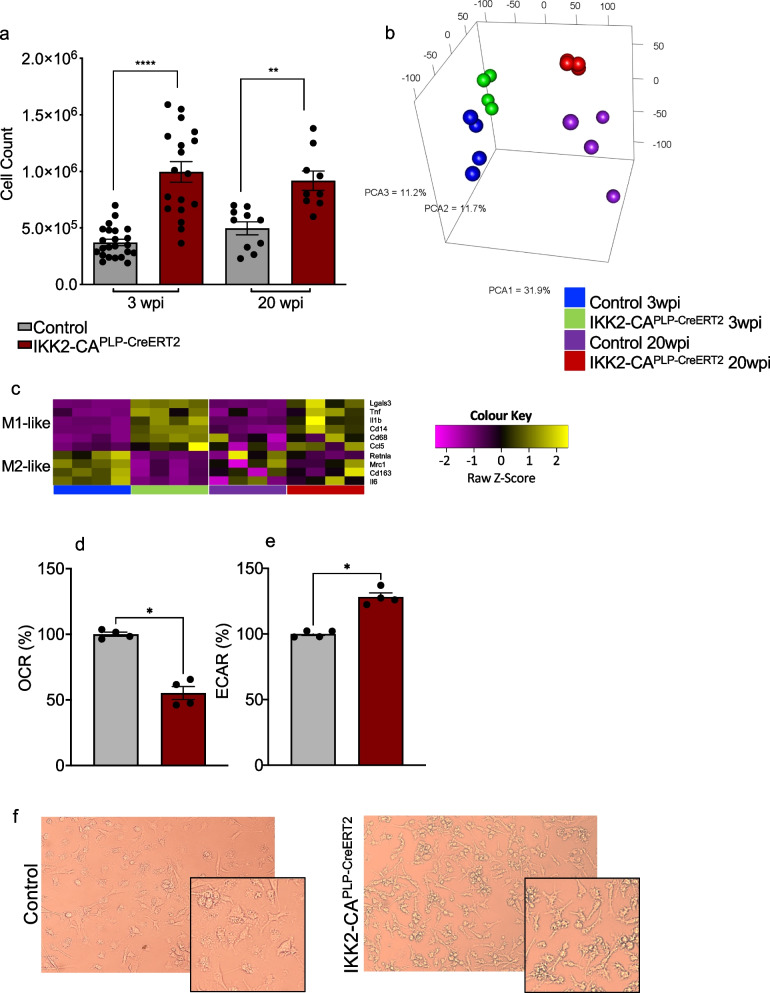
Chronic NF-κB activation in OLs triggers non-cell-autonomous expansion and activation of microglia cells. **a** Cell number of isolated Cd11b^+^ microglia cells from IKK2-CA^PLP−CreERT2^ mice is significantly increased compared to Cd11b^+^ microglia cells derived from control mice at 3 and 20 wpi (3 wpi: Control: 0.37 ± 0.03 × 10^6^, IKK2-CA^PLP−CreERT2^: 1.3 ± 0.24 × 10^6^ *n* = 20–23, 20 wpi: Control: 0.5 ± 0.05 × 10^6^, IKK2-CA^PLP−CreERT2^: 0.85 ± 0.1 × 10^6^, *n* = 10–23, two brains with identical genotype were pooled and set to *n* = 1). **b** Principal component analysis determines distinct differential gene expression between control and IKK2-CA^PLP−CreERT2^ Cd11b^+^ microglia at 3 and 20 wpi. **c** IKK2-CA^PLP−CreERT2^ Cd11b^+^ microglia are activated towards an M1 pro-inflammatory phenotype. RNA Sequencing analysis reveals upregulation of M1-like and downregulation of M2-like marker genes in IKK2-CA^PLP−CreERT2^ Cd11b^+^ microglia compared to control Cd11b^+^ microglia as depicted in the heatmap. All deregulated genes shown reached significance at 3 wpi, while only *Lgals3, IL1β* and *Cd14* reached significance at 20 wpi. **d** and **e** Seahorse analysis of Cd11b^+^ microglia from IKK2-CA^PLP−CreERT2^ mice revealed decreased oxidative phosphorylation (D, OCR normalized to control; IKK2-CA^PLP−Cre−ERT2^: 55.2 ± 4.9%, *n* = 3–4) and increased glycolysis (E, ECAR normalized to control; IKK2-CA^PLPCreERT2^: 128.2 ± 3.1%, *n* = 3–4) 3 wpi. **f***Ex-vivo* analysis of primary Cd11b^+^ microglia revealed distinct morphological differences between control and IKK2-CA^PLP−CreERT2^ Cd11b^+^ microglia. IKK2-CA^PLP−CreERT2^ Cd11b^+^ microglia show a rather bushy, activated phenotype. Data are shown as mean ± SEM. Grey bars, control; red bars, IKK2-CA.^PLP−CreERT2^. Statistical analysis: Two-way ANOVA multiple comparison test followed by Bonferroni´s post hoc test, Two-tailed Mann–Whitney-U Test, (*p < 0.05; **p < 0.01; ***p < 0.001; ****p < 0.0001 NS, non-significant (*p* > 0.05))

We identified prominent reactive microgliosis (Fig. 1e and f) in our IKK2-CA^PLP−CreERT2^ model suggesting non-cell-autonomous effects upon NF-κB activation in OLs. Therefore, we purified Cd11b^+^ microglia cells next to the O4^+^ cell isolation (Fig. S4a and Fig. S6a) from the same mice, and detected a significant expansion of isolated microglia cells in IKK2-CA^PLP−CreERT2^ mice compared to control animals (Fig. 6a).

RNA-Sequencing analysis of purified CD11b^+^ microglia revealed 1282/1954 and 187/416 up- and downregulated genes at 3 and 20 wpi, respectively (Fig. S6b and c). PCA shows a distinct clustering of the different groups (Fig. 6b) and gene expression profiling points to an inflammatory polarization state [44] of IKK2-CA^PLP−CreERT2^ derived microglia (Fig. 6c). Interestingly, gene expression analysis reveals a prominent overlap with aged white matter-associated microglia (WAM; S6d) and also uncovers a cellular senescence signature in IKK2-CA^PLP−Cre^ microglia [45] (3 wpi, Fig. S6e). In line with this, IKK2-CA^PLP−CreERT2^ microglia (3 wpi) exhibit a decrease in oxidative phosphorylation and an increase in glycolytic activity in seahorse analysis (Fig. 6d and e), and developed a rather bushy shape morphology (Fig. 6f). While the cell count of isolated microglia stayed significantly elevated in IKK2-CA^PLP−CreERT2^ mice, gene expression gets dampened at 20 wpi arguing for quantitative and/or qualitative changes in paracrine signaling components released by IKK2-CA^PLP−CreERT2^ OLs. According to this, we identified several, potentially NG2-glia/OPC growth promoting factors markedly upregulated in IKK2-CA^PLP−CreERT2^ microglia like Cspg4, Pdgfa, Hgf1 and Nrp1 at 3 wpi, which are normalized again at 20 wpi (Table S5).

### Traumatic brain injury promotes senescence of oligodendrocytes and decline in MBP protein

**Fig. 7:**
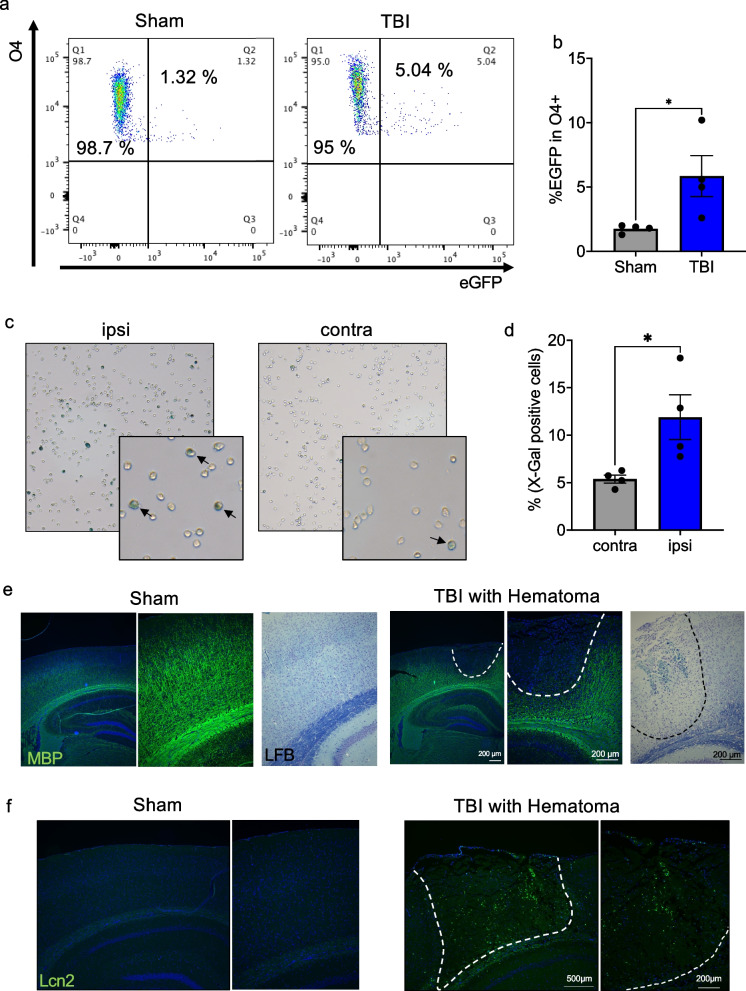
Traumatic brain injury induces senescence in OLs and myelin loss in the impact area. **a** and **b** NF-ĸB is activated in O4^+^OLs post TBI as determined by FACS analysis of O4^+^ OLs isolated from sham and TBI treated ĸ-EGFP reporter gene mice. A significant fraction of O4^+^ cells from TBI-treated ĸ-EGFP mice show eGFP expression 14 days post TBI (sham 1.75 ± 0.15%, TBI: 5.85 ± 1.58% *n* = 4). **c** and **d** A significant higher percentage of X-gal positive cells was detected in O4.^+^ OLs isolated from the ipsilateral hemisphere 14 dpi post TBI indicating senescence associated ß-galactosidase activity compared to uninjured contralateral hemisphere. Black arrows mark blue colored X-gal positive cells (contra: 5.38 ± 0.37%, ipsi: 11.89 ± 2.04%, *n* = 4). **e** Immunofluorescence and LFB analysis revealed strong loss of myelin basic protein (MBP) and reduced LFB staining within the insult area as indicated by dotted lines (3 dpi). **f** In contrast, the NF-ĸB target gene and SASP factor Lcn2 is found upregulated in the insult region as determined by immunofluorescence staining (3 wpi post TBI). Dotted lines indicate the TBI insult area. Data are shown as mean ± SEM. Grey bar, sham/contra; blue bar, TBI/ipsi. Statistical analysis: Two-tailed Mann–Whitney-U Test, (*p < 0.05; **p < 0.01; ***p < 0.001; ****p < 0.0001 NS, non-significant (*p* > 0.05))

To address the translational relevance of our findings, we focused on traumatic brain injury (TBI) as there is strong evidence from patients that TBI induces white matter lesions. However, it is not known whether PoMiCS of OLs contributes to WMD in TBI. To this end, we conducted experimental TBI, in particular the most common closed head injury (CHI), and performed bulk RNAseq analysis of insult tissue taken from the ipsilateral brain hemisphere of wildtype TBI and sham treated animals. PCA uncovered strong changes in gene expression in response to TBI (data not shown). Subsequent GO term analysis revealed an obvious overlap in differentially expressed genes previously found in O4^+^ OLs isolated from IKK2-CA^PLP−CreERT2^ mice and bulk insult tissue post TBI. These genes are associated with stress response, cellular senescence and SASP factors (Tables S1, S2 and S3). Interestingly, myelination-associated genes were slightly but significantly upregulated by TBI (Table S4) whereas isolated OLs rather show a mild downregulation. We then performed TBI and purified O4^+^ OLs from the ipsi- and contralateral side of NF-κB reporter gene mice [46] and subsequent FACS analysis confirmed NF-κB activation in OLs post TBI (Fig. 7a and b). Importantly, we identified a clear enrichment of SA-β-galactosidase positive OLs isolated from the ipsilateral side indicating TBI-induced senescence (Fig. 7c and d). Consistent with that immunohistology shows a downregulation of MBP in parallel to Lcn2 upregulation, a well-known NF-κB target gene and SASP factor (Fig. 7e and f). Thus, PoMiCS of OLs is a valid principle relevant for TBI pathogenesis and potentially acting in WMD.

## Discussion

Our study provides important insights how IKK/NF-κB signaling contributes to stress regulation in myelinating oligodendrocytes. We demonstrate that NF-κB in mature OLs is able to induce cell-autonomous and non-cell-autonomous stress reactions overall triggering WMD. NF-κB activation in OLs promotes microgliosis, astrogliosis and expansion of NG2-glia/OPCs leading to increased levels of OLs. Our data favor the hypothesis that age-associated white matter loss does not directly depend on the demise of OLs. Rather it is initiated by NF-κB-induced PoMiCS, which presents as a dysfunctional OL state characterized by deficits in myelin homeostasis. As an immediate consequence, steady-state myelinogenesis and/or myelin maintenance becomes impaired. In the long-term this culminates in white matter degeneration (graphical abstract).

Myelination and motor learning are mutually dependent on each other. Physiological myelination is indispensable for learning new motor tasks [37], whereas exercising of motor tasks is able to promote myelination or remyelination [47–49]. As a consequence, white matter defects come along with deficits in motor learning as the fine-tuned cortical ensemble becomes disturbed [50]. Our data indicate that chronic IKK/NF-κB signaling in a subset of CC1 + OLs is sufficient to impair myelin homeostasis and thereby forcing behavioral deficits. Concurrently, the increased proliferation of NG2-glia and the higher number of CC1 + OLs found in IKK2-CA^PLP−CreERT2^ mice argue for a ‘hypermyelination-like’ phenotype. This might represent a potential safeguard mechanism to replace dysfunctional, stressed or senescent OLs. Our RNA sequencing data show that NF-κB activation in OLs induces paracrine effects as differential clustering of IKK2-CA^PLP−CreERT2^ microglia was apparent in PCA. Furthermore, their gene expression profile reflects a rather pro-inflammatory M1-like signature, also known to arise in the context of CNS injury [51, 52]. We therefore consider it likely that microglia cells get instructed by NF-κB activated OLs to promote NG2-glia/OPC proliferation. Indeed, we identified several potentially NG2-glia/OPC growth promoting factors upregulated in IKK2-CA^PLP−CreERT2^ microglia like Cspg4, Pdgfa, Hgf1 and Nrp1. While Cspg4 (encoding Ng2) is upregulated in microglia upon acute brain injuries [53], Nrp1 (Neuropilin-1) was attributed to NG2-glia/OPC expansion in the white matter in an age-dependent manner, and its deletion was found to impair myelin homeostasis. Nrp1 can activate the platelet derived growth factor receptor alpha (Pdgfrα), similar to its consensus ligand, Pdgfa, to promote NG2-glia/OPC proliferation [54]. Hepatocyte growth factor (Hgf) was found to be secreted by microglia in spinal cord lesions in the remitting phase of experimental autoimmune encephalopathy [55].

Our results support a model where NF-κB activation in mature OLs induces a gene expression program including the release of secreted factors (SASP and others) which results in an injury like polarization program in microglia that is able to promote non-cell-autonomous NG2-glia/OPC proliferation. However, the additional OLs derived thereof might not be fully terminally differentiated and functionally integrated due to the lack of co-stimulating signals. Indeed, in brain injury, different factors like environmental enrichment and stimulation of neuronal activity were found to increase the integration capability of newly matured OLs [56–59]. Thus, the additional OLs in the IKK2-CA^PLP−CreERT2^ model arise from an activated backup system but rather stay dysfunctional due to the lack of an immediate need.

Recently, PoMiCS was postulated as a potential protective measure relevant for tissues like the CNS, possessing a high proportion of terminally differentiated post-mitotic cells [9]. The OL lineage represents a specific case as senescence might be applied to both mitotic NG2-glia/OPCs and post-mitotic OLs. Senescent NG2-glia/OPCs fail to differentiate into mature myelinating OLs thereby reducing the remyelination process at advanced age in mouse models of MS [60, 61]. Similar, senescent NG2-glia/OPCs were associated with Alzheimer´s Disease (AD) and treatment with senolytic drugs improved disease symptoms [62]. In contrast, aging of mature OLs seems to impair the maintenance of myelin sheaths, probably due to cellular PoMiCS [9, 63]. The fact, that OLs can accumulate DNA damage (e.g., by oxidative stress) thereby activating senescence markers like p53 and p16 supports the idea of stress-induced senescence in mature OLs [8, 9]. Importantly, a recent study provided comprehensive evidence that mature OLs are able to undergo a senescent program upon genetically induced DNA damage [64], supporting the critical role of PoMiCS to OL dysfunction.

Studies on NF-κB function in OLs mostly focused on remyelination efficacy in a disease context and importantly, only limited time windows of young ages were analyzed [65–67]. Inflammation was rather considered a consequence but not a cause of pathological demyelination [68, 69]. A different IKK2-CA mouse model (CNP/Cre x R26Stop^fl^ikk2ca) also shows that OLs’ viability is not affected by NF-κB but also did not impair myelination until the age of 8 weeks [70]. In contrast to our model, NF-κB activation in this study remained very low as typical NF-κB target genes were not significantly induced.

Our RNAseq analysis of O4^+^ OLs isolated from IKK2-CA^PLP−CreERT2^ mice clearly revealed gene expression profiles resembling an obvious NF-κB signature together with stress responses and cellular senescence. This includes classical NF-κB target genes, hallmarks of cellular senescence, and stress response markers indicative for ER stress, oxidative stress and DNA damage. OLs are exceptionally sensitive to oxidative stress and this vulnerability increases with age as adult-born OLs ensheath a higher number of different axonal segments [71]. O4^+^ sorted and ex vivo cultivated OLs from IKK2-CA^PLP−CreERT2^ mice showed an increased glycolytic activity and oxidative phosphorylation indicating a direct effect of NF-κB on OL metabolism. However, a scenario of vicious cycle formation is also conceivable whereat enhanced ROS formation due to increased oxidative phosphorylation accelerates stress responses. Consistent with our findings, senescent cells are known to undergo metabolic reprogramming to tailor their energy demand [72]. The detailed mechanisms how PoMiCS in OLs contributes to white matter degeneration upon aging is not resolved. A recent report [73] hypothesized that senescence in OLs might be independent of p16. Our RNA-Seq data revealed a clear upregulation of p16 and p21 in OLs derived from IKK2-CA^PLP−CreERT2^ mice. Oxidative stress (increased levels of 8-OHdG) and SA-β-Galactosidase activity were found *post mortem* [74] in human aged white matter tissue suggesting that PoMiCS is a valid concept possibly preceding white matter loss in humans. We also provide evidence that conditions known to result in progressive WMD like TBI, activates NF-κB and senescence in OLs. The molecular underpinnings of white matter changes in TBI are not well established so far. TBI induces multiple stress pathways which differ in quality (e.g., iron overload upon bleedings, edema formation) and quantity and may overlap only to a limited degree (e.g., oxidative stress, neuroinflammation). Therefore, aging-driven senescence and TBI-induced senescence processes most likely differ in quantity (and kinetics) as the expression level of relevant genes (e.g., Spp1, C3, Sepine1, Hmox1) evidently vary between the IKK2-CA^PLP−CreERT2^ model and TBI.

In addition to the persisting microgliosis, our RNA seq data argue for an abundance of a CD11b^+^/CD11c^+^ microglia population in IKK2-CA^PLP−CreERT2^ mice. This subset of microglia cells has been found enriched in the white matter and associated with aging-dependent accumulation of myelin debris [75]. Single cell RNA-sequencing identified prominent gene signatures associated with phagosome function, antigen processing and presentation compared to homeostatic microglia in this white matter associated microglia (WAM) [76]. WAM exhibit high *Trem2* expression, an important factor for phagocytosis of myelin debris [76, 77]. Importantly, this specific expression profile is even more pronounced in our model supporting the idea that NF-κB activation in OLs can initiate premature aging conditions in both OLs and microglia.

The role of stress response pathways in the regulation of myelination dynamics is controversially discussed. The integrated stress response (ISR) is a protective innate mechanism that can reduce the cytotoxic impact of aberrant inflammatory conditions. It is initiated via the phosphorylation of eIF2α (a subunit of the eIF2 protein complex) by different upstream kinases like PERK, finally repressing initiation of translation. Modulation of eIF2 activity has been shown to regulate the ISR and prolonging ISR through genetic or pharmacological approaches confirmed a protective role of eIF2α phosphorylation in experimental autoimmune encephalomyelitis [78–80]. We found that NF-κB activation in O4^+^ OLs induces a stress response similar to ISR including phosphorylation of eIF2α. This renders OLs in a senescent and dysfunctional state finally promoting WMD.

Our data reveal an important molecular link to vanishing white matter disease (VWMD), one of the most prevalent hereditary white matter diseases in childhood. Patients suffer from cerebral white matter degeneration associated with increased numbers of NG2-glia/OPCs similar to our IKK2-CA^PLP−CreERT2^ model. VWMD is caused by mutations in one of the five eukaryotic translation initiation factor genes, eucaryotic initiation factor 2B (eIF2B), which are involved in protein synthesis regulation [81]. The eIF2B complex functions as guanine nucleotide exchange factor (GEF) for eIF2 and VWMD loss-of-function mutations overall show a decrease in eIF2B GEF activity leading to repression of translation similar to eIF2α phosphorylation. Although eIF2B is a global regulator of protein translation, glia cells appear selectively vulnerable, as in VWMD brains of both mice and patients, ISR deregulation was observed in myelinating OLs and astrocytes.

Our findings provide an integrated mechanistic view how senescence of mature OLs can promote white matter loss under chronic neuroinflammatory conditions but also upon acute traumatic insults. Cell-type-specific modulation of NF-κB activity might open up new therapeutic strategies to reduce or delay age-dependent and post-traumatic WMD. Importantly, small molecule inhibitors of the ISR known to restore protein synthesis by counteracting p-eIF2α activity, might be attractive tools to interfere with PoMiCS of mature OLs in different clinical settings.

## Material and methods

### Transgenic mice

Mice were housed under specific standardized pathogen-free conditions in the animal facility at Ulm University. Food and water were provided ad libidum, a 12light/12dark cycle was obeyed. IKK2-CA^PLP−CreERT2^ was generated by crossing single transgenic Tg(Plp1-cre/ERT2)1Ueli [82] mice (short PLP-CreERT2) with mice single transgenic for Gt(ROSA)26Sor^tm4(Ikbkb)Rsky^ [83]. The RFP^PLP−CreERT2^ Cre reporter line was established by crossing single transgenic Tg(Plp1-cre/ERT2)1Ueli [82] mice with mice single transgenic for Gt(ROSA)26Sor^tm1Hjf^ [84]. The RFP^PLP−CreERT2^ model allows a direct in situ monitoring of recombination-dependent RFP expression i.e., cellular deletion efficiency. All mouse lines were bred on a C57BL/6 background. Activation of the genetic construct was conducted at the age of 9–13 weeks by 3 intraperitoneal (i.p.). injections of tamoxifen (2 mg tamoxifen dissolved in sun flower seed oil, Sigma-Aldrich, St. Louis, MO, USA) within 5 days and 14 days of tamoxifen enriched food (400 mg tamoxifen citrate/ 1 kg, Genobios, Laval, France), Fig. S1b. Thereafter animals were observed up until 40 weeks post induction (wpi). Wild-type and single transgenic littermates were used as control and just like the double transgenic animals subjected to tamoxifen administration, both male and female animals were included in the study.

All animal experiments were performed in compliance with the guidelines of the German Animal Protection Act and were approved by the Regierungspräsidium Tübingen.

### Behavioural analyses

#### Neurological Severity Score (NSS)

A 10-point NSS originally established to monitor neurological deficits after traumatic brain injury (TBI) was used to assess neurological deficits [34]. This scoring system consists of 10 tests, including tasks to measure cognitive and motor functions (e.g., beam walk, round-stick balance, exit circle, gait pattern, and exploratory interest in new environment), whereby 1 point is given for failure of the task and 0 points for succeeding. Thus, a maximum NSS of 10 points indicates severe neurological dysfunction, with failure of all tasks. In the present study, the NSS was assessed starting post induction at least every 4 weeks up to 40 wpi.

#### Rotarod

Motor behavior was analyzed with the ENV-575 M rotarod (Med Associates, St. Albans, VT, USA). After 1 min at 4 rpm for adjustment, the cylinder accelerated within 5 min to 40 rpm. The latency until falling off the accelerating rotarod was recorded. For the trained group, pretraining was assessed in the second week of tamoxifen food (consisting of 3 trials each on 3 consecutive days) and analysis was performed at least every 4 weeks up to 40 wpi. One group was left untreated and firstly admitted to pretraining after 20wpi.

#### Beam walking test

In this test, the mice had to traverse a narrow beam to escape from a small, elevated platform to a closed dark box, with subtle encouragement by the experimenter. For the trained group, a protocol with 4 training trials per day for 3 consecutive days with a 12 mm square beam (length 100 cm) was used and analysis was done in duplicates on different beam sizes (12 mm square, 5 mm square, 17 mm round, 11 mm round) at least every 4 weeks up to 40 wpi. The untrained group was only admitted to 1 day of trials on the 12 mm square beam 20wpi and analyzed on the different beam sizes thereafter.

#### Grip strength measurement

Grip strength was measured using a grip strength meter at maximum strength mode (Panlab, Harvard Apparatus); 3 trials were recorded and averaged. Grip strength was recorded at least every other week.

#### Ladderwalk

The ladder consists of two transparent plates (square: 69.5 × 15 cm) and cylindrical ladder rungs (8 cm long, Ø of 2 mm). The ladder walk has a length of 60 cm with a space of 1 cm between rungs for the regular ladderwalk and spaces varying between 0.5 and 2 cm for the irregular ladderwalk. Each mouse crossed the beam four times consecutively, while recorded with a Samsung NX1000 camera. The recorded videos were analyzed at ¼ of the original speed to count slips.

### Primary cell isolation

Primary OLs and microglia from adult mice were isolated using the Adult Brain Dissociation Kit, OctoMACS® with heaters, O4 and Cd11b beads as well as MS columns in a magnetic field (Miltenyi Biotec, Bergisch-Gladbach, Germany) according to the manufacturer’s protocol. In brief, brains were dissected, enzymatically digested, and debris was removed. The obtained cell suspension was labeled with O4 beads, washed, and then added to an MS column. The pellet of purified O4 positive OLs was snap-frozen in liquid nitrogen (N_2_) and stored at -80 °C until further analysis. The flowthrough and three washing volumes of the column containing unlabeled cells were combined and labeled again, this time with CD11b beads, washed and purified using an MS column. The pellet of purified CD11b positive microglia was snap-frozen in liquid nitrogen (N_2_) and stored at -80 °C until further analysis.

### X-Gal staining

Frehsly isolated primary O4 + OLs were seeded as a droplet of 300μL with a concentration of 1 × 10^6^ cells/mL in 0.5%BSA in PBS in the middle of a well in a 6-well plate. After cells were allowed to attach for 1.5 h at room temperature, the well was filled with 0.5%BSA in PBS very carefully before centrifuged at 200 g for 3 min at room temperature. Cells were then stained using the Senescence β-Galactosidase Staining Kit (Cell signaling #9860S) according to manufacturer´s instructions. In brief, cells were fixed for 10 min at room temperature and subsequently covered with staining solution and incubated at 37 °C for 24-48 h. If clear blue color formation was visible, cells were mounted with 70% glycerol. Images were taken with a Leica DM IRB Microscope (Wetzlar, Germany) and analyzed.

### Protein extraction and immunoblot analysis

Tissue samples from different brain regions were snap-frozen in liquid nitrogen, pulverized with a pestle, aliquoted, and stored at -80 °C. For protein extraction an aliquot of one spatula was lysed in 200 μL (approximately three times its volume) of KA-lysis buffer (25 mM Tris–HCl, 150 mM NaCl, 25 mM sodium pyrophosphate, 50 mM β-glycerophosphate, 50 mM NaF, 2 mM EGTA, 2 mM EDTA, 1 mM DTT, 10% glycerol, 1% Triton X-100 (pH 8.0)) supplemented with protease inhibitors (1 mM PMSF and CompleteMini Tablet; Roche Diagnostics, Mannheim, Germany). After centrifugation (30 min, 13,000 rpm), the supernatant was used as the total protein extract. Samples were denatured with a fourfold concentrated Laemmli buffer (200 mM Tris–HCl, 15% glycerol, 4% SDS, 5% β-mercaptoethanol, bromphenol blue). Cell pellets were lysed at a concentration of 10^7^ cells per 100 μL SDS sample buffer (62.5 mM Tris–HCl (pH 6.8), 2% SDS, 10% Glycerin, 50 mM DTT, 0.01% bromphenol blue) and sonicated 3 times for 5 s. Protein extracts from tissue samples as well as cell pellets were boiled for 5 min at 100 °C. Equal amounts of protein (50 μg of tissue samples and 25 μL of cell pellet samples) were separated by SDS-PAGE and transferred to nitrocellulose membranes via wet transfer. After blocking with 5% nonfat dry milk in TBS-T (0.05% Tween 20) buffer for 1 h at room temperature, primary antibodies (see below) were incubated in blocking solution overnight at 4 °C or for 2 h at room temperature. After 3 washing steps, membranes were incubated with horseradish peroxidase–coupled secondary antibody for 1 h at room temperature. Membranes were exposed to ECL detection reagent (Thermo Fisher Scientific, Waltham, MA, USA) and developed by ECL.

### Histology and immunostaining

Two different techniques were used: a) Brains were fixed by immersion with 4% paraformaldehyde (PFA) overnight at 4 °C, dehydrated, embedded in paraffin, and cut to 7 mm–thick coronal sections on a microtome (Microm HM355S; Thermo Fisher Scientific). For immunofluorescence, after rehydration, heat mediated antigen retrieval was performed with sodium citrate (10 mM, pH 6.0, 0.05% Tween 20) and the tissue sections were additionally incubated with 0.5% Triton X-100 for 30 min. Sections were washed with PBS and blocked with 5% bovine serum albumin (BSA) for 1 h at room temperature. Incubation with primary antibodies (in 5% BSA) was performed overnight at 4 °C and incubated afterward with secondary antibodies (in 5% BSA) for 1 h at room temperature with 100 ng/ml DAPI for nuclear counterstaining. For histology using Luxol Fast Blue (LFB) and Nissl staining, after dehydration sections were incubated in 0.1% LFB solution at 60 °C overnight, rinsed in 95% ethanol and distilled water before incubated in 0.05% Lithium carbonate solution for 5 s followed by two rinses in 70% ethanol for 10 s each. After washing in distilled water, staining was checked under the light microscope and the lithium carbonate incubation with following washing step repeated until a sharp contrast between grey and white matter was visible. Sections were then incubated in 0.1% cresyl violet staining solution for 4 min and thereafter again dehydrated using isopropyl alcohol and xylene and mounted Entellan (Sigma-Aldrich, St. Louis, MO, USA). b) PFA-fixed brain samples were obtained as previously reported [85]. Briefly, the animals were terminally anesthetized with ketamine and xylazine, transcardially perfused with a peristaltic pump (speed: 5 mL/min) by infusing 25 mL of ice-cold PBS followed by 50 mL of cold 4% PFA (pH 7.4). Brain samples were quickly dissected, post-fixed in 4% PFA for 18 h at 4 °C, washed in PBS, and dehydrated in 30% sucrose for 36 h. Samples were sectioned at -18 °C in a cryostat (Leica CM1950, Wetzlar, Germany) at a thickness of 30 μm. The sections were washed in PBS, blocked in PBS + 3% BSA + 0.5% Triton-X or PBS + 10% goatserum + 0.5% Triton-X and incubated with the appropriate antibody combination from 16–72 h at 4 °C, followed by washing in PBS (45 min × 3) and incubation with a corresponding combination of fluorophore-coupled secondary antibodies for 2 h at room temperature; after washing, sections were dried and mounted with Mowiol® (Carl Roth, Karlsruhe, Germany). For each experimental group or timepoint, at least three animals were processed and analyzed. Images were taken with an All-in-one fluorescence microscope BZ-X810 from Keyence (Neu-Isenburg, Germany) with a DAPI, FITC, TexasRed®, Cy5®, and a bright field filter, 10–40 × magnification, the BZ Viewer for capturing, and BZ Analyzer for merging. Images of stained sections were captured with equal exposure times per channel and the same graphical pre-processing within one experiment. For analysis and quantification, the ImageJ software (Rasband, W.S., ImageJ, U. S. National Institutes of Health, Bethesda, Maryland, USA) was used.

### Antibodies for immunoblotting and immunostaining

The following antibodies were used for immunoblot analysis: rabbit-anti-IKK2 (CST-2678, Cell Signaling, Danvers, MA, USA), rabbit-anti-p-p65 (CST-3033, Cell Signaling, Danvers, MA, USA), rabbit-anti-p65 (sc-372, Dallas, TX, USA), chicken-anti-GFP (ab13970, Cambridge, UK), rabbit-anti-tubulin (ab6046, Cambridge, UK), rabbit-anti-PLP1 (CST-28702, Cell Signaling, Danvers, MA, USA), rabbit-anti-MOG (CST-96457, Cell Signaling, Danvers, MA, USA), rabbit-anti-MBP (CST-2396, Cell Signaling, Danvers, MA, USA), rabbit-anti-GAPDH (CST-3686, Cell Signaling, Danvers, MA, USA), rabbit-anti-p-eIF2α (CST-3597, Cell Signaling, Danvers, MA, USA), rabbit-anti-eIF2α (CST-5324, Cell Signaling, Danvers, MA, USA), rabbit-anti-ERK2 (sc-1647, Dallas, TX, USA).

For immunofluorescence, the following primary antibodies were used: mouse-anti-CC1 (OP80, Merck, Darmstadt, Germany), mouse-anti-GSTπ (BD610719, BD, Franklin Lakes, NJ, USA), chicken-anti-GFP (GFP-1020, AvesLab, Davis, CA, USA), mouse-anti-GFAP (sc-33673, Dallas, TX, USA), rabbit-anti-NG2 (AB5320, Merck, Darmstadt, Germany), rabbit-anti-MBP (Biolegend 836,504, San Diego, CA, USA), mouse-anti-Neurofilament-H (SMI32P) (Biolegend 801,701, San Diego, CA, USA), rabbit-anti-ß3-tubulin (Biolegend 802,001, San Diego, CA, USA), rabbit- anti RFP (abcam ab124754, Cambridge, UK).

### RNA extraction, cDNA synthesis, and qRT-PCR

Total RNA from tissue samples and cell pellets was isolated using the Peq-Gold Trifast Kit (Peqlab, Erlangen, Germany) or RNA-Solv® Reagent (Omega Bio-Tek, Norcross, GA, USA) as described in the manufacturer’s protocol. 1 μg of total RNA was used to synthesize cDNA with the Transcriptor High Fidelity cDNA Synthesis kit (Roche, Grenzach-Whylen, Germany) with oligo-dT-primers according to the manufacturer’s instructions. Quantitative PCR (qPCR) assays were run in the Lightcycler 480 Instrument (Roche, Grenzach-Whylen, Germany) with primers and hydrolysis probes designed by the Roche Universal Probe Library (UPL) system. As a reference gene, the housekeeping gene hypoxanthine–guanine phosphoribosyl transferase (Hprt) was used. Primer sequences and UPLs are available upon request.

### Electron microscopy

Brains were quickly dissected and fixed in 0.1% Glutaraldehyde (GA) (Agar Scientific, Stansted, UK), 4% PFA, 2% Sucrose in 0.1 M Sorensen Phosphate Buffer for 24 h. Area of interest (Corpus Callosum) was dissected sagittal and again fixed overnight in 2.5% GA, 0.1% Sucrose in 0.1 M Sorensen Phosphate Buffer. Samples were then washed in PBS and post-fixed in 2% osmium tetroxide in PBS. After dehydrating the samples in a graded series of isopropanol, they were blockstained in 2% uranyl acetate in ethanol and embedded in Epon. Semi-thin (500 nm) sections were stained with toluidine blue and analyzed using light microscopy to find the area of interest. Ultra-thin Sects. (80 nm) were cut on a microtome using a diamond knife (Leica EM UC7, Wetzlar, Germany) and collected on carbon coated formwar films on 200 mesh copper grids (Plano, Wetzlar, Germany) contrasted with 0.3% lead citrate for 1 min and imaged using a TEM 1400 (Jeol, Tokyo, Japan).

### Cellular respiration and extracellular acidification

Primary OLs and microglia (see above) were seeded in XF96 V3 PS cell culture Microplates (Agilent Technologies, Santa Clara, USA) coated with 0.01% Poly-D-Lysin (Thermo Fisher Scientific, Waltham, MA, USA) at a density of 60.000 cells/well and 50.000 cells/well, respectively. OLs were plated in the following medium: NeuroMacs Medium supplemented with 2% NeuroBrew-21 (Miltenyi Biotec, Bergisch-Gladbach, Germany), 1% penicillin/streptomycin, 0.5 mM Glutamine (Thermo Fisher Scientific, Waltham, MA, USA). For proliferation, 10 ng/mL PDGF-AA and 10 ng/mL FGF-2 (Peprotech, Hamburg, Germany) were added freshly to the medium. After two days, 2 nM Triiodothyronine (T3) (Sigma-Aldrich, St. Louis, MO, USA) was added for differentiation and furthermore every second day until measurement at day 8. Microglia were seeded in DMEM supplemented with 10% FBS, 1% penicillin/streptomycin, 2 mM Glutamine (Thermo Fisher Scientific, Waltham, MA, USA). The medium was exchanged every second day until measurement at day 8. On the day of measurement, the cells were washed with XF assay medium (Agilent Technologies, Santa Clara, USA) containing 1 mM sodium pyruvate and 2 mM glutamine and incubated in fresh XF medium for 1 h under CO_2_ free conditions. Thereafter, oxygen consumption and extracellular acidification rates (OCR and ECAR) were measured simultaneously using a Seahorse XFe96 Flux Analyzer (Agilent Technologies, Santa Clara, CA, USA). The following injections and final concentrations were used: glucose (10 mM), oligomycin (2 μM), carbonylcyanide-p-trifluoromethoxyphenylhydrazone (FCCP, 2 μM) and antimycin A (0.5 μM)/ rotenone (0.5 μM). Data were normalized to JanusGreen as a surrogate for cell number per well.

### Rna-sequencing analysis

Total RNA extraction was performed as stated above, library construction and RNA-sequencing were conducted by Novogene (London, UK). Messenger RNA was purified from total RNA using poly-T oligo-attached magnetic beads. After fragmentation, the first strand cDNA was synthesized using random hexamer primers, followed by the second strand cDNA synthesis using dUTP for directional library. The library was checked with Qubit and real-time PCR for quantification and bioanalyzer for size distribution detection. Quantified libraries were pooled and sequenced on Illumina platforms, according to effective library concentration and data amount. The clustering of the index-coded samples was performed according to the manufacturer’s instructions. After cluster generation, the library preparations were sequenced on an Illumina platform and paired-end reads were generated. Raw data (raw reads) of FASTQ format were firstly processed through fastp. In this step, clean data (clean reads) were obtained by removing reads containing adapter and poly-N sequences and reads with low quality from raw data. At the same time, Q20, Q30 and GC content of the clean data were calculated. All the downstream analyses were based on the clean data with high quality. Reference genome (GRCm38) and gene model annotation files were downloaded from genome website browser (NCBI/UCSC/Ensembl) directly. Paired-end clean reads were aligned to the reference genome using the Spliced Transcripts Alignment to a Reference (STAR) software, which is based on a previously undescribed RNA-seq alignment algorithm that uses sequential maximum mappable seed search in uncompressed suffix arrays followed by seed clustering and stitching procedure. STAR exhibits better alignment precision and sensitivity than other RNA-seq aligners for both experimental and simulated data. FeatureCounts was used to count the read numbers mapped of each gene. And then RPKM of each gene was calculated based on the length of the gene and reads count mapped to this gene. RPKM, Reads Per Kilobase of exon model per Million mapped reads, considers the effect of sequencing depth and gene length for the reads count at the same time, and is currently the most commonly used method for estimating gene expression levels [86].

### Ex-vivo magnetic resonance imaging

Magnetic resonance imaging (MRI) was performed on a dedicated ultrahigh field 11.7 T small animal system (BioSpec 117/16, Bruker Biospin, Ettlingen, Germany) equipped with a 9 cm gradient insert (BGA-S9) operating with ParaVision 6.01. All data were acquired using a cryogenically-cooled 1H two-element surface (MRI CryoProbe™, Bruker BioSpec, Ettlingen, Germany) transmit/receive coil. For 3D brain imaging: FLASH with acquisition parameters as follows: TR/TE = 55/7 ms, flip angle FA = 15°, matrix 300 × 224 × 244, Δr = 40 × 40 × 40 µm^3^). field-of-view FOV = 12 × 9 × 9.85 mm3, and bandwidth = 50 kHz using 18 signal averages without interpolation. TA: 14h40m. Sequence weighting was T1-weighted.

### Traumatic brain injury – Closed Head Injury model (CHI)

Twelve-week-old wild type or NF-κB reporter gene mice [46] were subjected to experimental CHI with a standardized weight-drop device [34, 87]. In brief, the animals were anesthetized with ketamine (Pfizer Pharma, Karlsruhe, Germany), with an i.p. dose of 100 mg/kg bodyweight, and 2% xylazine (Bayer Health Care, Monheim, Germany), with an i.p. dose of 16 mg/kg body weight Afterward, the skull was exposed by a longitudinal incision of the skin, and a focal blunt injury was induced in the left hemisphere by dropping a 330 g metal rod on the skull from a height of 2.7 cm. After trauma, the mice received supporting oxygenation with 100% O_2_, the wound was sutured, and the animals were placed into a warmed recovery cage with ad libitum access to food and water. Buprenorphine analgesia (Temgesic; Essex Pharma, Munich, Germany) was administered subcutaneously (0.03 mg/kg body weight) immediately after trauma and every 8 h thereafter until 24 h.

### Statistical analysis

Statistical analyses were performed with Prism software (GraphPad, San Diego, CA, USA) and are indicated in the specific figure legend. One- or 2-way ANOVA with Bonferroni’s correction was used to compare independent measurements at one or different time points, respectively. For nonparametric analysis Mann–Whitney U was used. All data are shown as mean ± SEM, statistical significance was set at *P* < 0.05.

## Supplementary Information

**Additional file 1: Supplementary Figure 1.** Functional characterization of the IKK2-CA^PLP-CreERT2^ mouse model. **a** Scheme of the conditional transgenic IKK2-CA^PLP-CreERT2^ mouse model. Expression of a constitutive-active version of IKK2 (IKK2-CA) and the IRES driven eGFP reporter gene is inhibited through a stopper cassette until removed by tamoxifen-activated Cre recombinase. Cre recombinase expression under control of the PLP promotor ensures specificity to mature OLs. **b **Transgene expression was induced by 3 intraperitoneal injections of tamoxifen (2 mg) followed by tamoxifen-enriched food (400mg/kg) for 14 days. **c** and **d **Immunohistochemical analysis of RFP^PLP-CreERT2^ mice revealed no leakiness of the transgenic system. Control and uninduced RFP^PLP-CreERT2^ animals (no tamoxifen treatment) revealed no RFP expression, while recombination efficiency in RFP^PLP-CreERT2^ animals was found at 63.2%±4.8% (*n* = 3). **e** Immunoblot analysis of midbrain and cerebellar tissue samples isolated from control and IKK2-CA^PLP-CreERT2^ mice at 3 wpi. Transgene activation was demonstrated by prominent IKK2 and eGFP expression, abundance of p-p65 validates functional activation of IKK/NF-κB signaling (*n* = 3). **f** Elevated Ccl5 gene expression levels in cortex, CC, cerebellar and spinal cord tissue 3 wpi indicate an inflammatory milieu within the CNS (*n* = 3-4). **g** NF-κB target genes iNos, IL1b and Lcn2 are prominently upregulated in the OL-rich corpus callosum whereas expression is unchanged in spinal cord, cortex and cerebellum. **h **and **i **Immunohistochemical analysis of Ki67+/Pdgfrα+ double positive cells revealed a significantly increased number of proliferating precursor cells. (3 wpi – Control (*n* = 3): 8.3±4.3 cells/mm^2^, IKK2-CA^PLP-CreERT2^ (*n* = 3): 30.8±3.2 cells/mm^2^; 20 wpi – Control (*n* = 3): 5.0±3.6 cells/mm^2^, IKK2-CA^PLP-CreERT2^ (*n* = 3): 10.1±3.8 cells/mm^2^). **j** and **k** Expression of the IKK2-CA transgene is restricted to mature OLs. Arrows indicate no colocalization of the GFP reporter gene with NG2 staining marking progenitor cells (cortex tissue, 3wpi). (I) Sciatic nerve tissue does not show GFP reporter gene expression in control or IKK2-CA^PLP-CreERT2^ animals. As positive control sciatic nerve tissue from a Sox10-GFP reporter mouse was used, which shows robust expression of GFP in Schwann cells. Data are shown as mean ± SEM. Grey bars, control; red bars, IKK2-CA^PLP-CreERT2^. Statistical analysis: One-way or Two-way ANOVA multiple comparison test followed by Bonferroni´s post hoc test (*p < 0.05; **p < 0.01; ***p < 0.001; ****p < 0.0001 NS, non-significant (*p* > 0.05)). **Supplementary Figure 2.** Consequences of IKK2-CA activation in OLs. **a **Weight development was observed over the time course of 40 weeks. IKK2-CA^PLP-CreERT2^ mice show normal weight gain until 19 wpi, thereafter natural weight gain is decreased in both male and female mice. **b** IKK2-CA^PLP-CreERT2^ mice do not lose pre-learnt motoric skills. If animals were introduced to the rotarod task immediately after transgene activation, performance of IKK2-CA^PLP-CreERT2^ and control animals was comparable over the entire time course of 40 weeks. **c** and **d **IKK2-CA activation in oligodendrocytes does affect cerebellar homeostasis. Measurement of cerebellar width showed no differences between IKK2-CA^PLP-CreERT2^ and control animals. The black line in D indicates the measured length (*n* = 5-7). **e **Motoric deficits do not issue from purkinje cell loss. Histochemical analysis using Nissl staining of the cerebellum shows intact morphology and normal purkinje cell number as indicated by black arrows. **f **Immunohistochemical analysis using SMI32 did not reveal axonal pathology in IKK2CA^PLPCreERT2^ animals. As a positive control tissue slides from TBI treated wildtype animals were used. Data are shown as mean ± SEM. Black dots/grey bars, control; red dots/bars, IKK2-CA^PLP-CreERT2^. Statistical analysis: Two-way ANOVA multiple comparison test followed by Bonferroni´s post hoc test, Two-tailed Mann-Whitney-U Test (*p < 0.05; **p < 0.01; ***p < 0.001; ****p < 0.0001 NS, non-significant (*p* > 0.05)). **Supplementary Figure 3.** NF-ĸB activation in OLs leads to white matter loss in the corpus callosum. **a **and **b Ex-vivo **MRI analysis of the brain at 20 wpi reveals reduced signal strength (appearing brighter) in the CC as well as reduction in width of the CC of IKK2-CA^PLP-CreERT2^ mice. White arrows indicate the CC. **c** Quantification of the width of the CC (*n* = 3-4). **d** and **e **Distribution of the axon diameter of myelinated axons in the CC is not changed IKK2-CA^PLP-CreERT2^ and control animals at 3 wpi and 20 wpi. **f **Diameter of unmyelinated axons depicted as single values reveals a greater scattering of the axon diameter of unmyelinated axons in the CC of IKK2-CA^PLP-CreERT2^ mice 20 wpi. **g** – **i **Luxol Fast Blue (LFB) staining revealed a significant decrease in the width of the CC in IKK2-CA^PLP-CreERT2^ animals at different bregma points at 20 wpi. Bregma points evaluated were Bregma -1mm (g), Bregma (h) and Bregma+1mm (i). **j **Quantification of g – i (Bregma-1mm: 3wpi: Control: 348±21.7 μm, IKK2-CA^PLP-CreERT2^: 344±16.8 μm; 20wpi: Control: 442±17.3 μm, IKK2-CA^PLP-CreERT2^: 353±16.2 μm; Bregma: 3wpi: Control: 231±8.9 μm, IKK2-CA^PLP-CreERT2^: 225±2.3 μm; 20wpi: Control: 235±3.1 μm, IKK2CA^PLP-CreERT2^: 184±13.0 μm; Bregma+1mm: 3wpi: Control: 508±40.6 μm, IKK2CA^PLPCreERT2^: 498±32.9 μm; 20wpi: Control: 624±3.9 μm, IKK2-CA^PLP-CreERT2^: 503±22.5 μm *n* = 3-4). Data are shown as mean ± SEM. Black lines/grey bars, control; red lines/bars, IKK2-CA^PLP-CreERT2^. Statistical analysis: Two-way ANOVA multiple comparison test followed by Bonferroni´s post hoc test, (*p < 0.05; **p < 0.01; ***p < 0.001; ****p < 0.0001 NS, non-significant (*p* > 0.05)). **Supplementary Figure 4.** Isolation of O4^+^ OLs from brains of IKK2-CA^PLP-CreERT2^ mice and subsequent transcriptome analysis. **a** Schematic workflow of the consecutive isolation of primary O4^+^ OLs and CD11b^+^ microglia from adult mouse brains. After tissue dissociation, the obtained cell suspension is treated with magnetic beads labeled with O4 antibodies thus allowing separation of O4^+^ OLs in a magnetic field. The remaining cell suspension is again treated with magnetic beads, this time labeled with Cd11b antibodies allowing isolation of Cd11b^+^ microglia in a second step. **b** Cell number of isolated O4^+^ OLs does not significantly differ between IKK2-CA^PLP-CreERT2^ and control animals at 3 and 20 wpi (3 wpi: Control: 0.90±0.05 x10^6^, IKK2-CA^PLP-CreERT2^: 0.81±0.05 x10^6^ *n* = 20-23, 20 wpi: Control: 1.1±0.07 x10^6^, IKK2-CA^PLP-CreERT2^: 1.3±0.09 x10^6^, *n* = 10-23, two brains with identical genotype were pooled and set to *n* = 1). **c **and **d **qRT-PCR reveals strong upregulation of the IKK2 transgene (IKK2-CA) as well as GFP reporter gene in O4^+^ OLs from IKK2-CA^PLP-CreERT2^ animals, **e** western blot analysis indicates robust expression of IKK2-CA protein solely in IKK2-CA^PLP-CreERT2^ O4^+^ OLs. Cortex tissue from IKK2-CA^PLP-CreERT2^ mice served as positive control (*n* = 3). **f **FACS analysis of cells isolated with O4^+^ beads shows a strong enrichment (70%) of O4^high^/CD11b^low^ cells. GFP expressing IKK2-CA^PLP-CreERT2^ O4^+^ OLs are strongly enriched within the isolated O4^+^ OL cell population. Quantification of the GFP+ O4^+^ cells 3 and 20 wpi (3 wpi: 27.8±4.1 % *n* = 9, 20 wpi: 9.2±1.5 % *n* = 5). **g** FACS staining of O4^+^ OLs with GalC and MOG as markers for intermediate and mature OLs revealed strong enrichment of myelinating OL within the bead-sorted O4 fraction without differences between control and IKK2-CA^PLP-CreERT2^ O4^+^ OLs (GalC: Control: 70.74±1.64 %, IKK2-CA^PLP-CreERT2^: 72.73±1.80 %; MOG: Control: 56.14±3.20 %, IKK2-CA^PLP-CreERT2^: 58.85±4.04 %, *n* = 3-5). **h** and **i** Volcano plot depicting upregulated and downregulated genes in O4^+^ OLs from IKK2CA^PLP-CreERT2^ mice compared to control mice at 3 and 20 wpi, revealed by RNA sequencing analysis. **j** and **k** RNA sequencing reveals significantly enriched NF-ĸB target gene expression (**i**) in IKK2-CA^PLP-CreERT2^ O4^+^ OLs whereas genes connected to the GO class myelin sheath formation (GO: 0043209) (**j**) are only slightly deregulated as 3 and 20 wpi (as indicated in the heatmaps). Out of the genes depicted only *Mobp *and *Plec *were found significantly deregulated at 3 and 20 wpi, while at 3 wpi additionally *Tppp, Plp1 *and *Msn *reached significance, as well as *Tubb4b, Calm1 *and *Gsn *at 20 wpi. Data are shown as mean ± SEM. Grey bars, control; red bars, IKK2-CA^PLP-CreERT2^. Statistical analysis: Two-tailed Mann-Whitney-U Test, (*p < 0.05; **p < 0.01; ***p < 0.001; ****p < 0.0001 NS, non-significant (*p *> 0.05)). **Supplementary Figure 5.** Gene enrichment analysis of O4+OLs with activated IKK/NFκB signaling. **a **and **b **GSEA shows strong enrichment in gene sets of cellular senescence (**a**) and p53 signaling pathway (**b**) in IKK2-CA^PLP-CreERT2^ O4^+^OLs compared to control O4^+^OLs at 3 wpi. **c **Heatmap depicts strong significant overall upregulation of SASP factors in IKK2CA^PLPCreERT2^ O4^+^OLs compared to control O4^+^OLs both at 3 and 20 wpi. **d **Schematic overview of translational arrest by phosphorylation of eucaryotic initiation factor 2, subunit alpha (eIF2α). Stress-activated kinases (e.g., PERK, PKR, GCN2 and HRI) are able to phosphorylate eIF2α leading to the inhibition of GDP to GTP exchange resulting in global translational arrest. **e **RNA sequencing analysis reveals enrichment in proteasome (MMU03050) in IKK2CA^PLPCreERT2^ O4^+^OLs 20 wpi. **Supplementary Figure 6.** NF-ĸB activation in OLs initiates an aging-associated microglia phenotype. **a** Bead-Isolated Cd11b^+^ microglia were subjected to FACS analysis using Cd11b-PE labeled antibody and revealed a purity of 83%. **b** and **c **Volcano plot depicting upregulated and downregulated genes in Cd11b^+^ microglia from IKK2-CA^PLP-CreERT2^ mice compared to control mice at 3 and 20 wpi (determined by RNA sequencing analysis). **d **Cd11b^+^ microglia isolated from IKK2-CA^PLP-CreERT2^ animals (3 wpi) show an overlap with aged white matter associated microglia (WAM) in gene sets involved in phagocytic activity and metabolism (Safaiyan 2021). IKK2-CA^PLP-CreERT2^ derived Cd11b^+^microglia exhibit a more pronounced elevation of genes related to lipid-metabolism and phagosome activity compared to WAM and activated microglia. Genes linked to ribosomal subunits and mitochondrial genes are rather downregulated in WAM IKK2-CA^PLP-CreERT2^ Cd11b^+^microglia. **e** GSEA uncovers gene signatures of cellular senescence in IKK2-CA^PLP-CreERT2^ Cd11b^+^ microglia compared to control microglia at 3wpi.

**Additional file 2:** **Table S1.** Differentially regulated genes associated with stress response, comparison of RNA Seq analysis of O4+OL isolated from IKK2-CAPLP-CreERT2 mice 3 wpi and TBI insult area bulk tissue. **Table S2.** Differentially regulated genes associated with Cellular Senescence, comparison of RNA Seq analysis of O4+OL isolated from IKK2-CAPLP-CreERT2 mice 3 wpi and TBI insult area bulk tissue. **Table S3.** Differentially regulated genes encoding for SASP Factors, comparison of RNA Seq analysis of O4+OL isolated from IKK2-CAPLP-CreERT2 mice 3 wpi and TBI insult area bulk tissue. **Table S4.** Differentially regulated genes associated with Myelin Sheath (GO Term: 0043209), comparison of RNA Seq analysis of O4+OL isolated from IKK2-CAPLP-CreERT2 mice 3 wpi and TBI insult area bulk tissue. **Table S5.** Differentially regulated genes in microglia associated with NG2-glia/OPC proliferation, RNA Seq analysis of Cd11b+ microglia cells isolated from IKK2-CAPLP-CreERT2 mice 3 and 20 wpi.

## Data Availability

All data generated in this study are included in this published article. Further RNA-Sequencing data will be made available to the public prior to publishing. All custom scripts are available from the corresponding authors upon reasonable request.

## Abbreviations

8-OHdG: 8-Hydroxyguanosine
AD: Alzheimer´s disease
CHI: Closed head injury
CNS: Central nervous system
DAMP: Damage-associated molecular patters
ECAR: Extracellular acidification rate
eIF2: Eucaryotic initiation factors 2
FACS: Fluorescence-activated cell sorting
GEF: Guanidine exchange factor
GFAP: Glial fibrillary acidic protein
GFP: Green fluorescent protein
GSEA: Gene set enrichment analysis
Iba1: Ionized calcium-binding adapter molecule 1
IKK: Inhibitor of nuclear factor kappa-B kinase
IKK2-CA: Constant active IKK2 transgene
ISR: Integrated stress response
LFB: Luxol fast blue
MBP: Myelin basic protein
MOG: Myelin oligodendrocyte glycoprotein
MRI: Magnetic resonance imaging
MS: Multiple sclerosis
NF-κB: Nuclear factor ‘kappa-light-chain-enhancer’ of activated B-cells
NSS: Neurological severity score
OCR: Oxygen consumption rate
OL: Oligodendrocyte
OPC: Oligodendroyte progenitor cells
PLP: Proteolipid protein
PoMiCS: Post mitotic cellular senescence
SA: Senescence associated
SASP: Senenscence associated secretory phenotype
TBI: Traumatic brain injury
TUNEL: TdT-mediated dUTP-biotin nick end labeling
VWMD: Vanishing white matter disease
WAM: White matter associated microglia
WMD: White matter degeneration
wpi: Weeks post induction
