# Supplementary material for: NF-κB is a critical mediator of post-mitotic senescence in oligodendrocytes and subsequent white matter loss
Source: Mol Neurodegener. 2023 Apr 17;18:24. doi: 10.1186/s13024-023-00616-5 (PMC10108549; doi:10.1186/s13024-023-00616-5)

Supplementary Figure 1

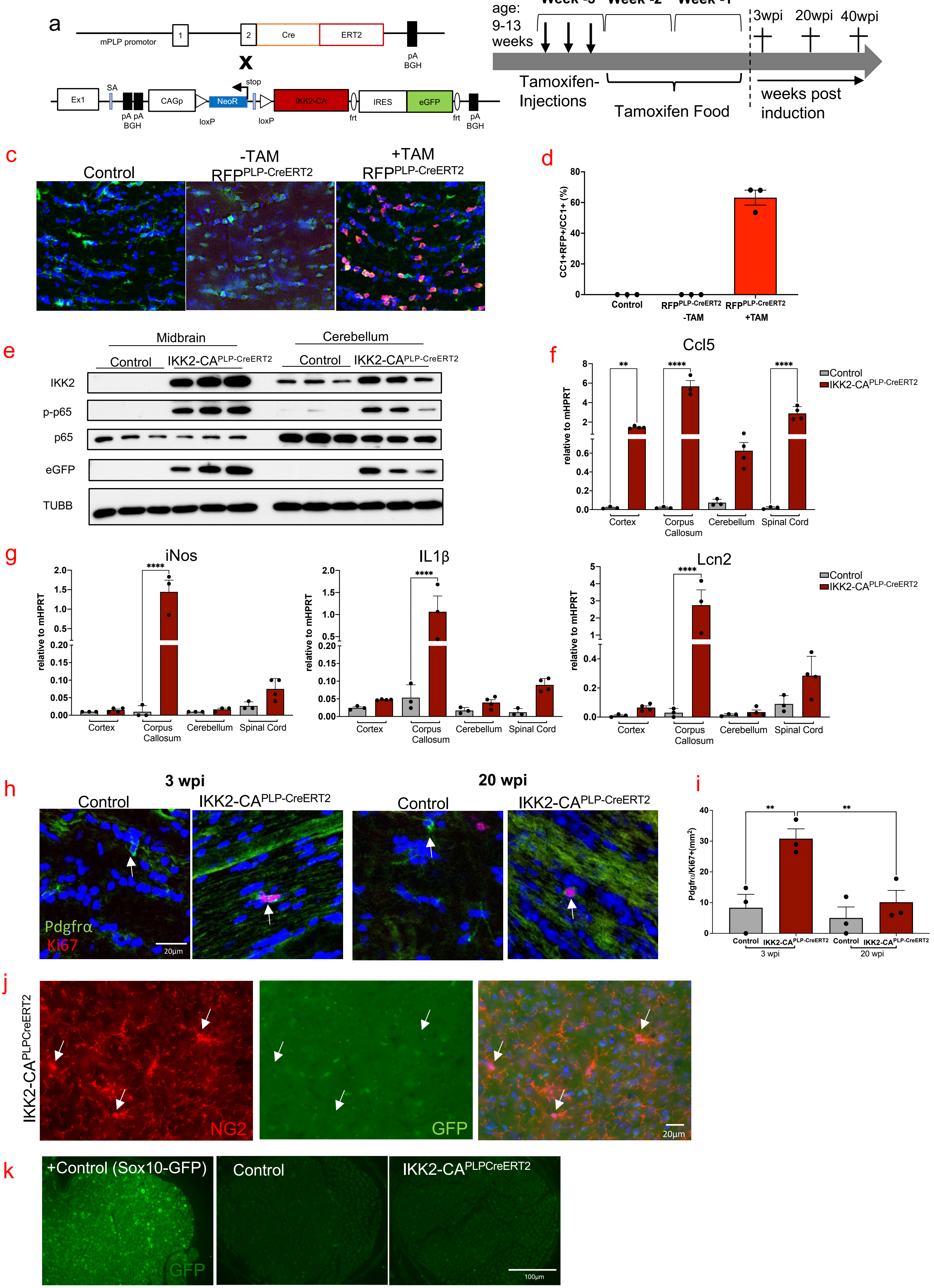

Supplementary Figure 2

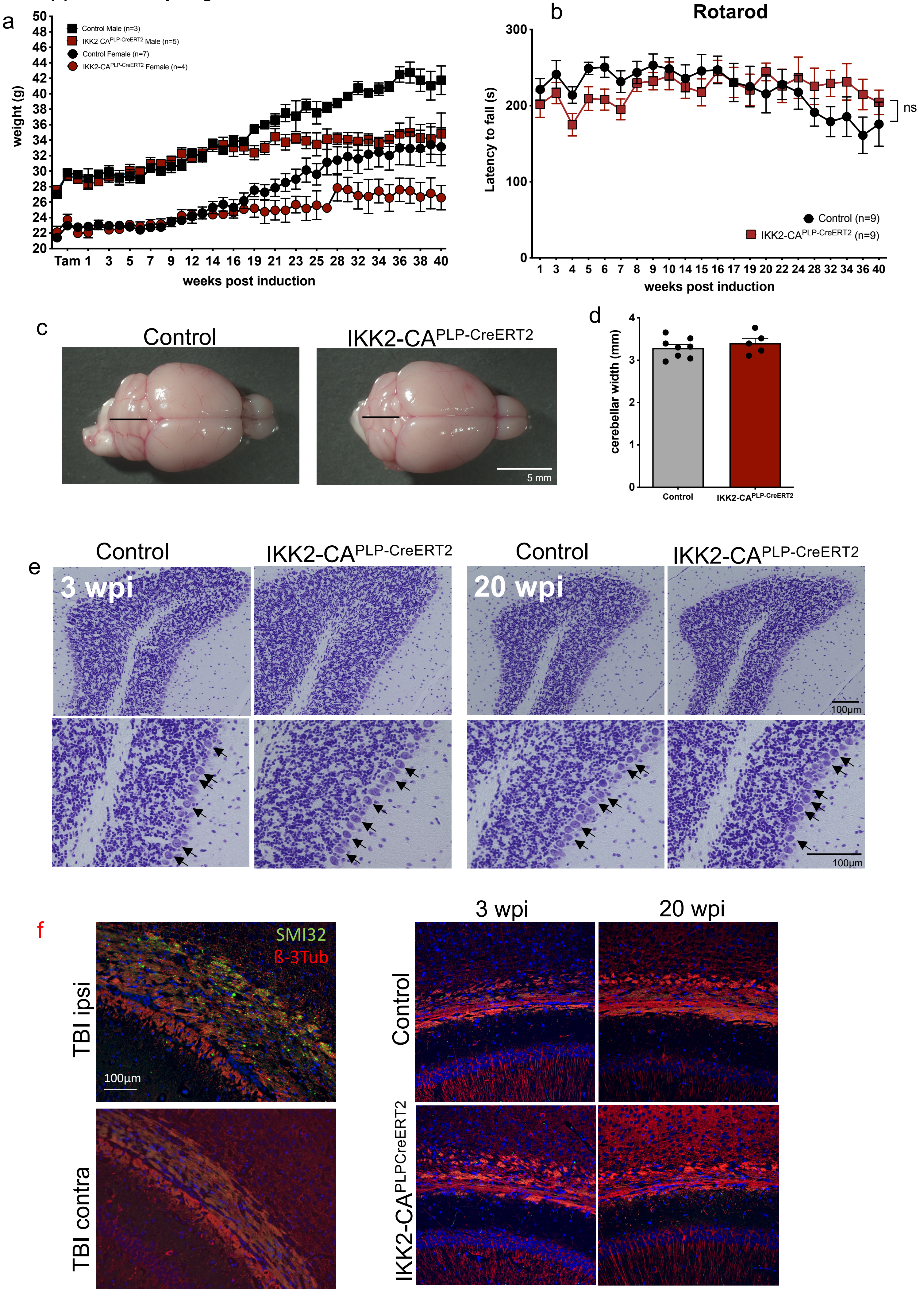

Supplementary Figure 3

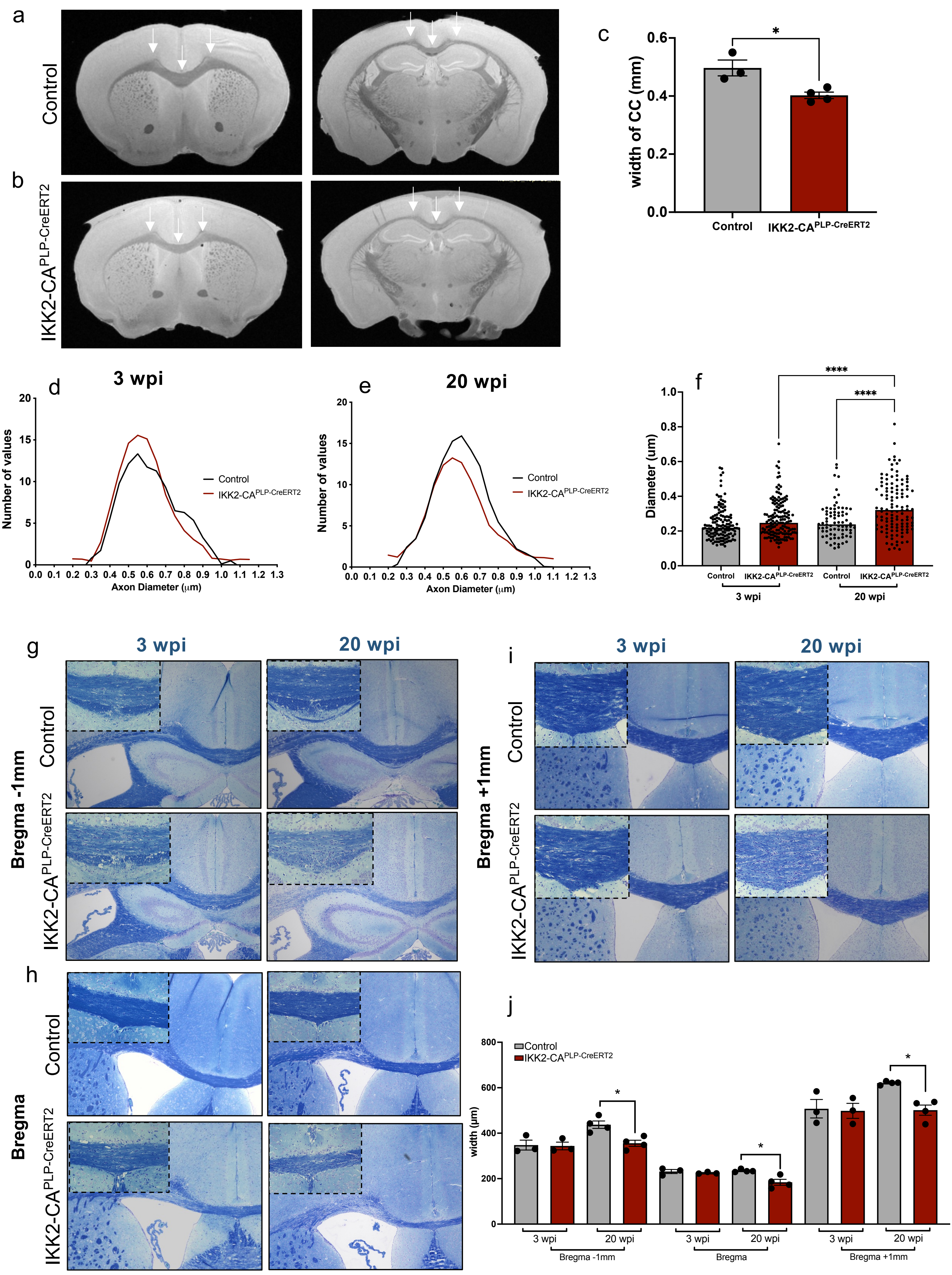

Supplementary Figure 4

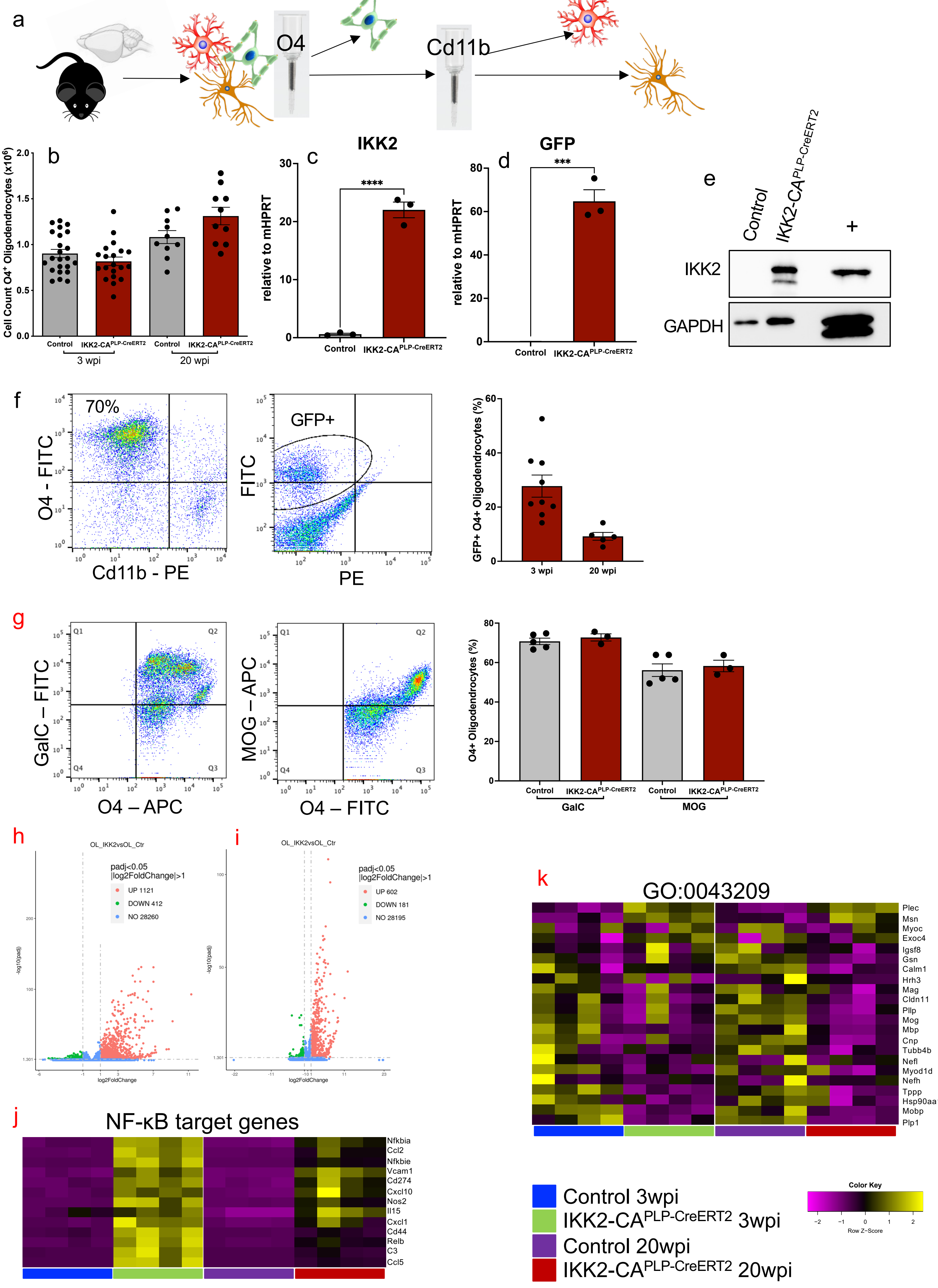

Supplementary Figure 5

a

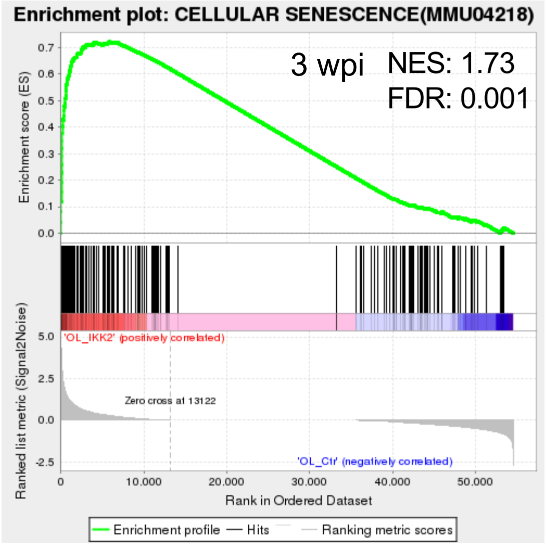

b

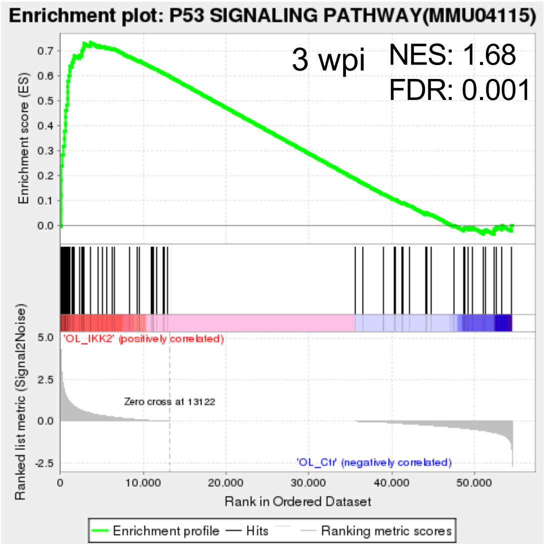

c

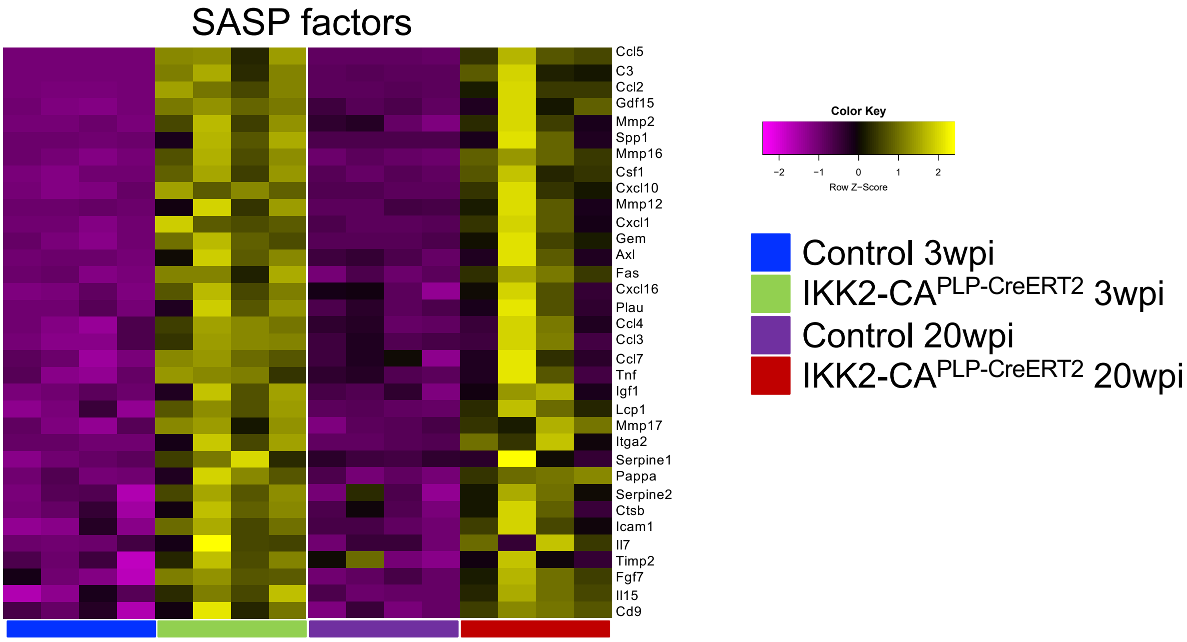

d

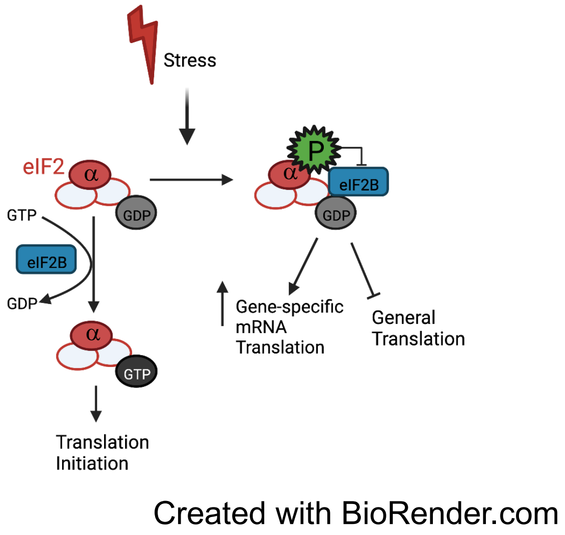

e

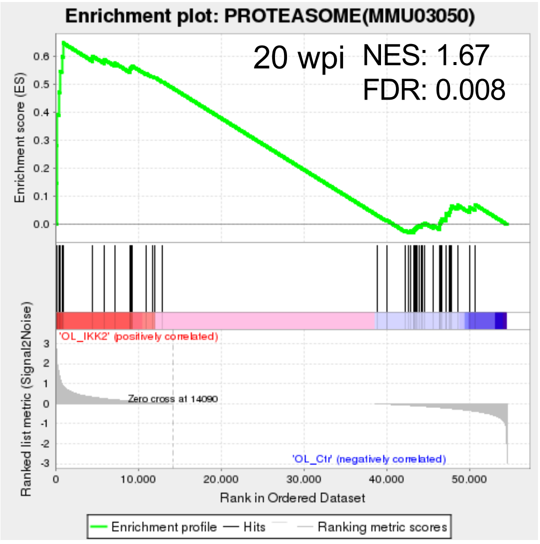

Supplementary Figure 6

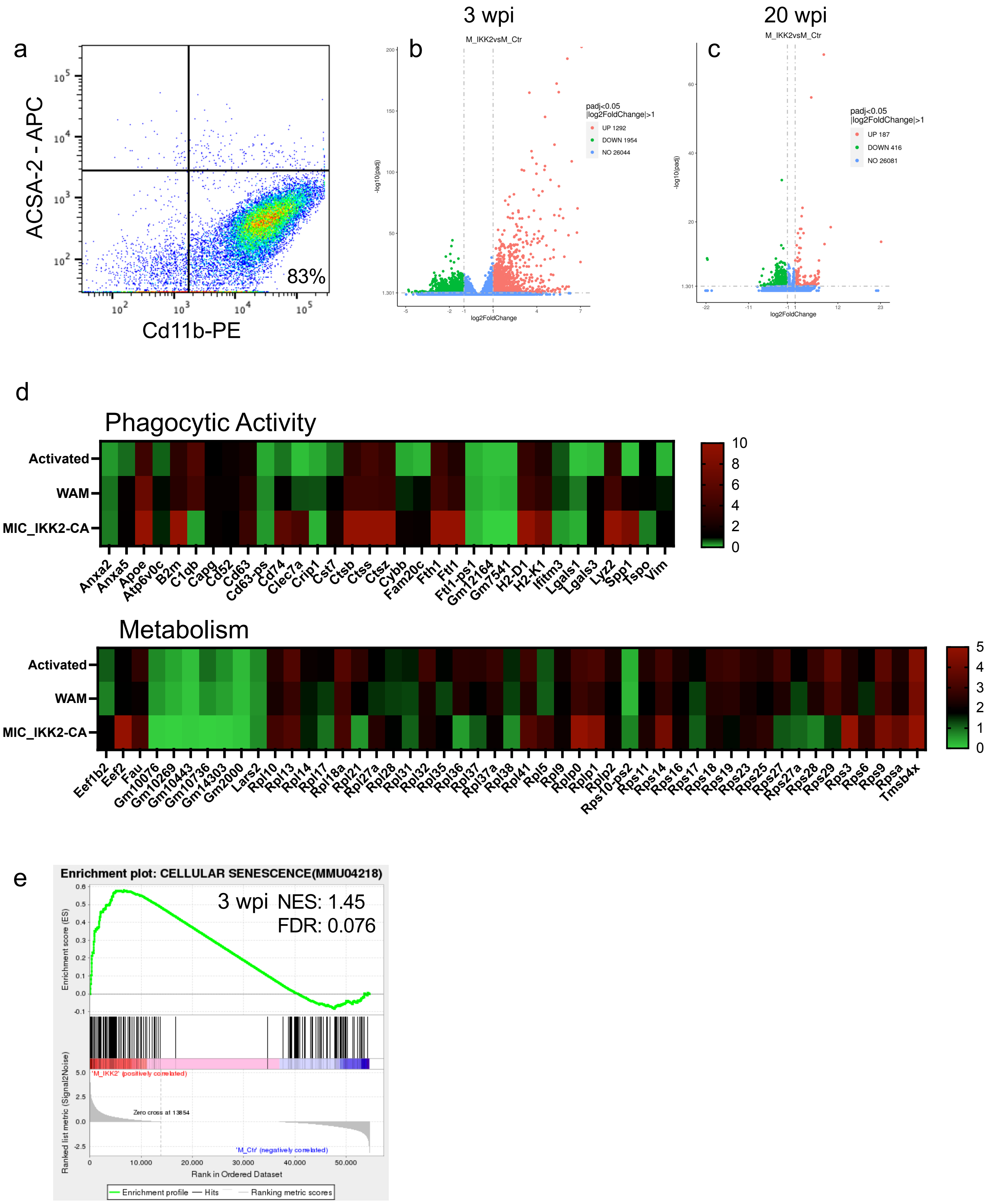

Supplement: Supplementary file 1 — Additional file 1: Supplementary Figure 1. Functional characterization of the IKK2-CAPLP-CreERT2 mouse model. a Scheme of the conditional transgenic IKK2-CAPLP-CreERT2 mouse model. Expression of a constitutive-active version of IKK2 (IKK2-CA) and the IRES driven eGFP reporter gene is inhibited through a stopper cassette until removed by tamoxifen-activated Cre recombinase. Cre recombinase expression under control of the PLP promotor ensures specificity to mature OLs. b Transgene expression was induced by 3 intraperitoneal injections of tamoxifen (2 mg) followed by tamoxifen-enriched food (400mg/kg) for 14 days. c and d Immunohistochemical analysis of RFPPLP-CreERT2 mice revealed no leakiness of the transgenic system. Control and uninduced RFPPLP-CreERT2 animals (no tamoxifen treatment) revealed no RFP expression, while recombination efficiency in RFPPLP-CreERT2 animals was found at 63.2%±4.8% (n = 3). e Immunoblot analysis of midbrain and cerebellar tissue samples isolated from control and IKK2-CAPLP-CreERT2 mice at 3 wpi. Transgene activation was demonstrated by prominent IKK2 and eGFP expression, abundance of p-p65 validates functional activation of IKK/NF-κB signaling (n = 3). f Elevated Ccl5 gene expression levels in cortex, CC, cerebellar and spinal cord tissue 3 wpi indicate an inflammatory milieu within the CNS (n = 3-4). g NF-κB target genes iNos, IL1b and Lcn2 are prominently upregulated in the OL-rich corpus callosum whereas expression is unchanged in spinal cord, cortex and cerebellum. h and i Immunohistochemical analysis of Ki67+/Pdgfrα+ double positive cells revealed a significantly increased number of proliferating precursor cells. (3 wpi – Control (n = 3): 8.3±4.3 cells/mm2, IKK2-CAPLP-CreERT2 (n = 3): 30.8±3.2 cells/mm2; 20 wpi – Control (n = 3): 5.0±3.6 cells/mm2, IKK2-CAPLP-CreERT2 (n = 3): 10.1±3.8 cells/mm2). j and k Expression of the IKK2-CA transgene is restricted to mature OLs. Arrows indicate no colocalization of the GFP reporter gene with NG [file 13024_2023_616_MOESM1_ESM.pdf]
