# Supplementary material for: NF-κB is a critical mediator of post-mitotic senescence in oligodendrocytes and subsequent white matter loss
Source: Mol Neurodegener. 2023 Apr 17;18:24. doi: 10.1186/s13024-023-00616-5 (PMC10108549; doi:10.1186/s13024-023-00616-5)
Supplement: Supplementary file 2 — Additional file 2: Table S1. Differentially regulated genes associated with stress response, comparison of RNA Seq analysis of O4+OL isolated from IKK2-CAPLP-CreERT2 mice 3 wpi and TBI insult area bulk tissue. Table S2. Differentially regulated genes associated with Cellular Senescence, comparison of RNA Seq analysis of O4+OL isolated from IKK2-CAPLP-CreERT2 mice 3 wpi and TBI insult area bulk tissue. Table S3. Differentially regulated genes encoding for SASP Factors, comparison of RNA Seq analysis of O4+OL isolated from IKK2-CAPLP-CreERT2 mice 3 wpi and TBI insult area bulk tissue. Table S4. Differentially regulated genes associated with Myelin Sheath (GO Term: 0043209), comparison of RNA Seq analysis of O4+OL isolated from IKK2-CAPLP-CreERT2 mice 3 wpi and TBI insult area bulk tissue. Table S5. Differentially regulated genes in microglia associated with NG2-glia/OPC proliferation, RNA Seq analysis of Cd11b+ microglia cells isolated from IKK2-CAPLP-CreERT2 mice 3 and 20 wpi. [file 13024_2023_616_MOESM2_ESM.docx]

Table S1

| **Fold Change_OL** | **Gene** | **Fold Change_TBI** |
| --- | --- | --- |
| 2.15 | **Hmox1** | 53.55 |
| 2.63 | **Atf3** | 14.93 |
| 8.17 | **Mmp2** | 3.61 |
| 1.27 | **Nfe2l2** | 2.61 |
| 1.54 | **Rad51** | 2.43 |
| 2.31 | **Brca1** | 2.42 |
| 1.66 | **Gpx1** | 2.15 |
| 1.72 | **Txn1** | 1.35 |
| 1.55 | **Txnrd1** | 1.30 |
| 2.70 | **Sesn2** | 1.21 |
| 1.63 | **Ppp1r15a** | 1.15 |
| 1.48 | **Atf4** | 1.06 |
| 1.67 | **Srxn1** | 0.98 |

All listed genes were significantly (p ≤ 0.05) deregulated, (n=5-6).

Table S2

| **Fold Change_OL** | **Gene** | **Fold Change_TBI** |
| --- | --- | --- |
| 2.71 | **Serpine1** | 40.33 |
| 2.24 | **Cdk1** | 26.80 |
| 6.69 | **Cdkn2a** | 14.14 |
| 1.87 | **Rbl1** | 3.64 |
| 8.36 | **Igfbp3** | 3.13 |
| 2.83 | **Eif4ebp1** | 2.82 |
| 3.19 | **Ccnd1** | 2.39 |
| 1.66 | **Nfkb1** | 1.68 |
| 1.72 | **Trp53** | 1.62 |
| 1.96 | **Mapk12** | 1.44 |
| 6.67 | **Gadd45b** | 1.43 |
| 1.95 | **Gadd45a** | 1.34 |
| 5.63 | **Cdkn1a** | 1.14 |
| 2.06 | **Capn1** | 1.09 |
| 1.93 | **Ccne1** | 0.96 |
| 1.98 | **E2f1** | 0.93 |
| 14.17 | **Mapk11** | 0.82 |

All listed genes were significantly (p ≤ 0.05) deregulated, (n=5-6).

Table S3

| **Fold Change_OL** | **Gene** | **Fold Change_TBI** |
| --- | --- | --- |
| 62.19 | **Spp1** | 226.34 |
| 30.94 | **Ccl2** | 42.14 |
| 2.71 | **Serpine1** | 40.33 |
| 3.44 | **Ccl3** | 28.38 |
| 3.28 | **Ccl4** | 25.00 |
| 19.93 | **Mmp12** | 21.82 |
| 2.98 | **Ccl7** | 20.37 |
| 10.43 | **Cxcl1** | 19.65 |
| 4.49 | **Cxcl16** | 19.65 |
| 6.77 | **Cxcl10** | 18.98 |
| 76.14 | **C3** | 14.62 |
| 4.45 | **Tnf** | 10.64 |
| 3.26 | **Lcp1** | 9.64 |
| 564.12 | **Ccl5** | 8.08 |
| 12.90 | **Gdf15** | 6.99 |
| 4.91 | **Plau** | 3.78 |
| 8.17 | **Mmp2** | 3.61 |
| 2.08 | **Ctsb** | 2.83 |
| 3.23 | **Csf1** | 2.28 |
| 7.13 | **Fas** | 2.21 |
| 15.30 | **Il7** | 2.10 |
| 2.02 | **Fgf7** | 2.00 |
| 2.44 | **Icam1** | 1.77 |
| 2.02 | **Il15** | 1.73 |
| 6.56 | **Itga2** | 1.70 |
| 7.18 | **Axl** | 1.46 |
| 4.81 | **Gem** | 1.35 |
| 1.69 | **Cd9** | 1.32 |
| 2.74 | **Serpine2** | 1.27 |
| 1.65 | **Timp2** | 1.19 |
| 4.57 | **Igf1** | 1.04 |
| 7.54 | **Pappa** | 0.90 |
| 2.90 | **Mmp17** | 0.80 |
| 7.57 | **Mmp16** | 0.68 |

All listed genes were significantly (p ≤ 0.05) deregulated, (n=5-6).

Table S4

| **Fold Change_OL** | **Significance** | **Gene** | **Fold Change_TBI** | **Significance** |
| --- | --- | --- | --- | --- |
| 1.88 | * | **Msn** | 5.39 | * |
| 1.10 |  | **Gsn** | 2.23 | * |
| 0.90 |  | **Cldn11** | 1.76 | * |
| 0.90 |  | **Pllp** | 1.56 | * |
| 0.87 |  | **Mog** | 1.39 | * |
| 0.95 |  | **Hrh3** | 1.27 | * |
| 0.85 |  | **Cnp** | 1.25 | * |
| 0.66 | * | **Mobp** | 1.24 | * |
| 0.92 |  | **Mag** | 1.17 | * |
| 0.65 | * | **Plp1** | 1.13 | * |
| 0.85 |  | **Mbp** | 1.12 | * |
| 0.83 |  | **Tubb4b** | 1.11 | * |
| 1.17 |  | **Myoc** | 1.10 | * |
| 0.77 |  | **Myo1d** | 1.07 | * |
| 1.14 |  | **Igsf8** | 1.03 | * |
| 2.64 | * | **Plec** | 0.99 | * |
| 0.75 |  | **Nefh** | 0.90 | * |
| 0.98 |  | **Calm1** | 0.85 | * |
| 0.71 |  | **Hsp90aa1** | 0.83 | * |
| 1.14 |  | **Exoc4** | 0.83 | * |
| 0.71 | * | **Tppp** | 0.82 | * |
| 0.80 |  | **Nefl** | 0.72 | * |

Only genes marked with * were significantly (p ≤ 0.05) deregulated, (n=5-6).

Table S5

| **Fold Change**  **Microglia 3 wpi** | **Significance** | **Gene** | **Fold Change**  **Microglia 20 wpi** | **Significance** |
| --- | --- | --- | --- | --- |
| 16.60 | * | **Cspg4** | 0.90 |  |
| 3.82 | * | **Dlk2** | 0.03 |  |
| 3.66 | * | **Hgf** | 3.51 |  |
| 2.84 |  | **Tacr1** | 0.09 |  |
| 2.31 | * | **Nrp1** | 0.88 |  |
| 2.30 | * | **Pdgfa** | 0.91 |  |
| 1.36 |  | **Jag1** | 1.04 |  |

Only genes marked with * were significantly (p ≤ 0.05) deregulated, (n=5).
